# Evaluation of the Influence of *Tanacetum vulgare* Extract on Cognitive Functions and Hippocampal BDNF Expression

**DOI:** 10.3390/molecules29235723

**Published:** 2024-12-04

**Authors:** Borislava Lechkova, Michaela Shishmanova-Doseva, Niko Benbassat, Reneta Gevrenova, Pepa Atanassova, Nadya Penkova, Lyudmil Peychev, Petar Hrischev, Zhivko Peychev, Stanislava Ivanova

**Affiliations:** 1 Department of Pharmacognosy and Pharmaceutical Chemistry, Faculty of Pharmacy, Medical University of Plovdiv, 4002 Plovdiv, Bulgaria; borislava.lechkova@mu-plovdiv.bg (B.L.); niko.benbasat@mu-plovdiv.bg (N.B.); 2 Research Institute, Medical University of Plovdiv, 4002 Plovdiv, Bulgaria; mihaela.shishmanova@mu-plovdiv.bg; 3 Department of Pharmacology, Toxicology and Pharmacotherapy, Faculty of Pharmacy, Medical University of Plovdiv, 4002 Plovdiv, Bulgaria; lyudmil.peychev@mu-plovdiv.bg; 4 Department of Pharmacognosy, Faculty of Pharmacy, Medical University-Sofia, 2 Dunav Str., 1000 Sofia, Bulgaria; rgevrenova@pharmfac.mu-sofia.bg; 5 Department of Anatomy, Histology and Embryology, Faculty of Medicine, Medical University of Plovdiv, 4002 Plovdiv, Bulgaria; pepa.atanasova@mu-plovdiv.bg (P.A.); nadya.penkova@mu-plovdiv.bg (N.P.); 6 Department of Physiology, Faculty of Medicine, Medical University of Plovdiv, 4002 Plovdiv, Bulgaria; petar.hrischev@mu-plovdiv.bg; 7 Department of Medical Informatics, Biostatistics and E-Learning, Faculty of Public Health, Medical University of Plovdiv, 4002 Plovdiv, Bulgaria; zhivko.peychev@mu-plovdiv.bg

**Keywords:** *Tanacetum vulgare*, cognitive functions, anxiolytic activity, BDNF, hippocampus

## Abstract

*Tanacetum vulgare* L. has been traditionally applied as a remedy for headaches, rheumatism, digestion, respiratory and neurological problems, and other medical conditions. However, the literature data on its effects on cognitive function are scarce. The aim of the present work was to evaluate the effects of two doses of *T. vulgare* alcohol extract on cognitive functions, hippocampal brain-derived neurotrophic factor (BDNF) expression, and organ toxicity in rats. Rats were treated with *T. vulgare* 200 mg/kg (TV 200) and 1000 mg/kg (TV 1000) for 28 days. After one week of pre-treatment, the animals were subjected to a series of tests. We found that in the active avoidance test, only TV 200 improved learning and memory, while in the passive avoidance test, both doses facilitated these processes. In addition, the two doses enhanced spatial memory. In the elevated plus maze test, only the higher dose of TV 1000 resulted in anxiolytic-like behavior. Both doses of the extract significantly increased the hippocampal expression of BDNF. We suggest that increased neurotrophic factor expression could be one of the important mechanisms underlying the cognition-enhancing effects of *T. vulgare* extract.

## 1. Introduction

The Asteraceae (Compositae) family is one of the largest plant families, consisting of more than 1600 genera and 25,000 species, a number of which have considerable medicinal significance [1]. Among these, the *Tanacetum* genus is the third largest and accounts for approximately 160 species, well distributed in Europe, Asia, and North America [2]. Its name stems presumably from the Greek word *athanasia* (meaning immortality), since tansies were used for embalming in the medieval period [3]. Historically, *Tanacetum* species have been utilized for medicinal, cosmetic, agricultural, and culinary purposes [4].

**Figure 1:**
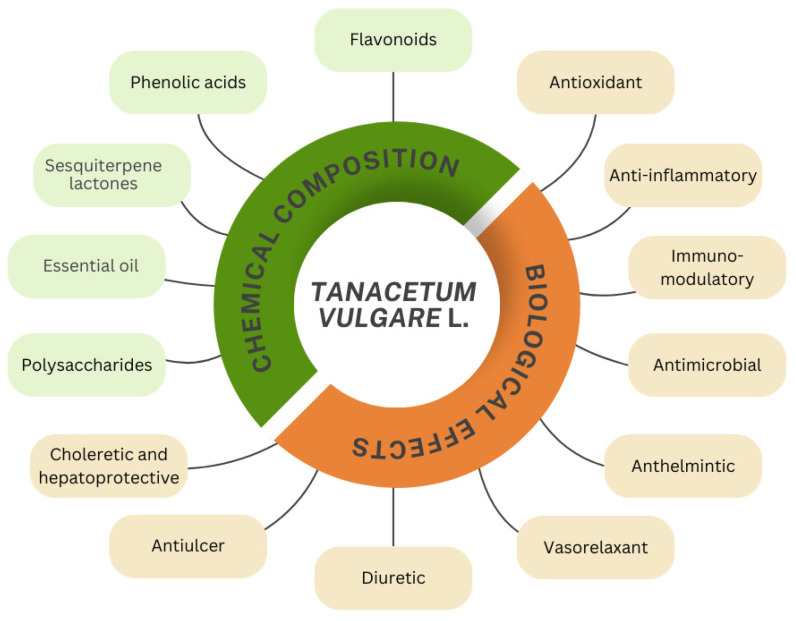
Chemical composition and biological effects of *T. vulgare* L.

*Tanacetum vulgare* (common tansy), one of the representatives of the genus, is a perennial plant reaching up to 150 cm in height, native to Europe and Asia, and later introduced to North America. The leaves are alternate, petiolate, divided compound. The flowering heads are yellow-golden, consisting of more than 100 individual florets [5,6]. It has been traditionally applied as a remedy for headache, rheumatism, diabetes, digestion, respiratory and neurological problems, wounds, and helminth infections [4,7,8]. Modern studies focused on determining its phytochemical composition established this aromatic plant as a rich source of both primary and secondary metabolites, which condition a broad spectrum of biological activities (Figure 1). The primary metabolites (carbohydrates, proteins, lipids) are vital for its growth, development, and reproduction, whereas the secondary ones are not directly required for the survival of the plants. Secondary metabolites include phenolics, terpenoids, and nitrogen-containing compounds (alkaloids) that are synthesized only in some plant species, playing a role in the adaptation to environmental factors and in plant defense. They can be used in plant classification as a taxonomic marker, and many of them have also been applied in the food, cosmetic, pharmaceutical, and agricultural industries [9,10].

Polysaccharides isolated from tansy flowers exhibit immunomodulatory effects [11], and Vislobokov et al. observed a membranotropic effect on transmembrane ionic currents of neurons of *Lymnaea stagnalis* [12]. According to Paderin et al., pectin isolated from the plant led to reduced feeding time and food intake in mice [13].

Regarding the composition of the *T. vulgare* essential oil, significant variation has been discovered due to environmental and genetic reasons [14]. Different chemotypes are distinguished by the presence of one compound whose concentration exceeds 40% (mono-chemotype) [15], or in the cases of two to three dominant terpenoids, mixed chemotypes have also been reported [16]. Major constituents are mono- and sesquiterpenes, e.g., α-thujone, β-thujone, camphor, chrysanthenol, chrysanthenyl acetate, 1,8-cineole, artemisia ketone, sabinene, myrtenol, and germacrene-D [17,18,19,20].

Nitric oxide (NO) is a free radical produced by three isoforms of the enzyme nitric oxide synthase (NOS)—neuronal (nNOS), inducible (iNOS), and endothelial NOS (eNOS) [21]. The overproduction of NO can trigger inflammation and oxidative and nitrergic stress, associated with neuronal toxicity and neurodegeneration [22,23]. *T. vulgare* essential oil inhibits NO production and exhibits anti-inflammatory and antioxidant effects [24]. Additionally, it shows activity against *Escherichia coli*, *Enterobacter cloacae*, *Staphylococcus aureus*, and fungi [3,24]. Synergism with different classes of antibiotics was displayed against some Gram-positive and Gram-negative clinically relevant bacterial strains [25]. Anthelmintic properties of tansy have been linked to both the presence of thujone and phenolic compounds [26].

Based on the content of sesquiterpene lactones, Todorova et al. indicated the existence of three chemotypes—germacranolide, eudesmanolide and lactone free [27]. Eudesmanolides isolated from flowers of *T. vulgare* from Sicily induce cytotoxic effects against cancer and healthy cell lines [28]. Ethyl acetate extract from aerial parts and isolated parthenolide (a germacranolide) exhibit anti-herpes virus activity [29]. Moreover, Schinella et al. suggested parthenolide was the main constituent responsible for the anti-inflammatory properties of tansy against 12-*O*-tetradecanoylphorbol 13-acetate-induced mouse-ear oedema [30].

In general, *Tanacetum vulgare* extracts contain phenolic compounds, most notably phenolic acids and flavonoids. Formerly reported are caffeoylquinic acids, rosmarinic acid, apigenin, luteolin, casticin, rutin, quercetin, and their glucosides and glucuronides [3,31,32,33,34]. The total phenolic content has been associated with the demonstrated antioxidant properties of the plant [32,35,36]. The methanolic extract of tansy aerial parts and isolated 3,5-*O*-dicaffeoylquinic acid showed high antioxidant activity in a study by Habtemariam et al., which supports the traditional use of the species as a wound healing agent, in rheumatoid arthritis, and other inflammatory diseases [37]. Moreover, the imbalance between the generation and the neutralization of free radicals in the organism plays a major role in several other illnesses such as cancer, diabetes, atherosclerosis, malaria, cataracts, bronchial asthma, and neurodegeneration [38,39].

Cognitive impairment may occur as a result of neurodegenerative diseases such as Alzheimer’s disease, Parkinson’s disease, and Huntington’s disease or due to aging, brain injuries, and the intake of some drugs. These represents a significant health problem of the 21st century, with a constant increase in the number of patients suffering from them, impacting their quality of life negatively and placing a burden on the health system. Various mechanisms are linked to the pathophysiology of these diseases—oxidative stress, inflammation, neurotoxicity, etc. [40,41,42,43]. In addition, a growing number of studies strongly suggest that decreased hippocampal brain-derived neurotrophic factor (BDNF) expression also contributes to the pathogenesis of these neurodegenerative disorders, leading to learning deficits and long-term memory loss [44,45]. BDNF belongs to the family of neurotrophins, which are endogenous proteins and important regulators of brain development, neuronal maturation, and neuroplasticity. BDNF plays a crucial role in synaptic activity and in the maintenance of cellular homeostasis [44,45]. The neurotrophic factor facilitates long-term potentiation in neurons, leading to a long-term increase in signaling transmission between them, which in turn plays a fundamental role in its cognition-enhancing effects [46].

Modern medicine and therapy approaches are directed toward not only treating the symptoms but also preventing and slowing down their progression [43,47]. There is a heightened interest in discovering substances of natural origin and studying their mechanism of action—polyphenols, terpenes, alkaloids, polysaccharides, etc. [43,48,49,50]. Plant-based products are generally well tolerated and can be used alone or with other medicines [51].

A number of studies on *T. vulgare* also revealed vasorelaxant [52], diuretic [53], antiulcer [54], choleretic, and hepatoprotective properties [55]. However, the literature data on its effects on cognitive function are scarce. Hence, the aim of the present work was to evaluate the effect of two doses of *T. vulgare* alcohol extract on cognitive functions, hippocampal BDNF expression, and organ toxicity.

## 2. Results

### 2.1. Chemical Composition of Tanacetum vulgare Extract

The evaluation of the chemical profile was performed in two steps. Quantification of phenolic compounds was carried out by HPLC-UV analysis by a method previously reported by Krasteva et al. [56]. UHPLC-HRMS analysis followed, confirming the obtained results and providing more detailed data about the chemical profile of the extract.

#### 2.1.1. Quantification of Phenolic Compounds by HPLC-UV Analysis

Initially, an HPLC-UV screening for the presence of nine phenolic acids and six flavonoids was performed [56]. The method was previously developed and reported in many other studies [56,57,58,59]. The presence of four phenolic acids and three flavonoids was established. The results are presented in Table 1.

#### 2.1.2. UHPLC-HRMS Profiling

The UHPLC-HRMS analysis of the *T. vulgare* extract yielded the identification/annotation of 99 secondary metabolites based on retention times, elemental composition, accurate masses, fragmentation patterns, relative abundance of precursor and fragment ions, as well as comparison with reference standards and literature data (Table A1).

##### Hydroxybenzoic, Hydroxycinnamic Acids, and Their Derivatives

Based on comparison with reference standards, three hydroxybenzoic acids (**1**, **4** and **15**), a hydroxycinnamic acid (**16**), and quinic acid (**7**) were identified in the extract.

Compounds **6**, **11** and **13** shared the same [M−H]^−^ at *m*/*z* 341.0871 (Figure S1). From the fragment ions arising from the hexose cross-ring cleavages ^0.4^Hex (−60 Da), ^0.3^Hex (−90 Da), and ^0.2^Hex (−120 Da) caffeoyl-*O*-hexose (**11**) (sugar ester) were deduced. In contrast, the absence of these fragment ions suggested (**6**) and (**13**) as the corresponding glycosides caffeic acid-*O*-hexoside and its isomer.

The isobaric pair **9** and **12** ([M−H]^−^ at *m*/*z* 357.0827) were assigned to caffeoylgluconic acid and its isomer affording relevant fragment ions at *m*/*z* 195.0503 [gluconic acid−H]^−^, 177.0396 [gluconic acid−H−H_2_O]^−^, 129.0177 [gluconic acid−H−CH_2_O−2H_2_O]^−^, and 87.6073 [gluconic acid−H−C_3_H_8_O_4_]^−^.

##### Acylquinic Acids

Eight mono-, twenty-four di-, and one triacylquinic acid (AQAs) and one acylquinic acid dimer were identified or annotated in the tansy extract (Table A1, Figure S2). The annotation of AQAs was based on previously reported fragmentation patterns and diagnostic ions in the MS/MS spectra [60,61,62].

The retention times and fragmentation patterns of compounds **21** and **22** ([M−H]^−^ at *m*/*z* 353.087) were consistent with those of neochlorogenic and chlorogenic acid reference standards. In full MS at 4.12 min ions at *m*/*z* 353.088 and 707.183 were registered. The former was a deprotonated caffeoylquinic acid molecule, while the latter was a neutral caffeoylquinic acid bond to the deprotonated caffeoylquinic acid. Thus, caffeoylquinic acid dimer (**25**) was deduced from the typical ions at *m*/*z* 353.088, 191.055, 173.044, and 135.044.

Compounds **23** and **26** exhibited bases peak at *m*/*z* 163.039 [*p*CoA−H]^−^ and 193.0498 [FA−H]^−^ and were identified as 3-*p*-coumaroylquinic acid and 3-feruloylquinic acid, respectively. The same precursor ion [M−H]^−^ at *m*/*z* 515.119 was shared by **30**, **42**, **43**, and **44**. They presented prominent ions at *m*/*z* 353.088 and 191.055, aligning with the losses of a caffeoyl moiety, and were assigned to dicaffeoylquinic acids. Discrimination between the isomers was based on Clifford’s hierarchical key and the intensity of the ions at *m*/*z* 335, 179, and 173 [60]. The abundance of the fragment ion at *m*/*z* 173.045, for instance, suggested that the caffeoyl groups occupy vicinal positions in **42** and **44**. 4,5-dicaffeoylquinic acid (**44**) was deduced from the insignificant “dehydrated” ion at *m*/*z* 335, and **42** was identified as 3,4-dicaffeoylquinic acid, confirmed by comparison with reference standards. Compounds **37**, **39**, **40**, and **41** ([M−H]^−^ at *m*/*z* 677.172) afforded fragment ions, which were consistent with the loss of a hexose moiety, followed by two caffeoyl residues. Thus, they were assigned to dicaffeoylquinic acid-hexoside isomers. Compound **54** with [M−H]^−^ at *m*/*z* 677.1528 exhibited subsequent losses of three caffeoyl residues at *m*/*z* 515.1191, 353.0881 and 191.0554, indicating tricaffeoylquinic acid. The abundance of the fragment ions at *m*/*z* 179.0341, 173.0446, and 135.0438 suggested it was 3,4,5-tricaffeoylquinic acid [61]. Compounds **45**, **46**, **49**, and **50** shared [M−H]^−^ at *m*/*z* 499.125, **47**, **48**, **51**, **52**, and **53** at *m*/*z* 529.135, and **27**, **28**, **31**, **32**, **35**, **36**, and **38** at *m*/*z* 533.130, iondicating *p*-coumaroyl-caffeoylquinic acids, feruloyl-caffeolylquinic acids, and hydroxydihydrocaffeoyl caffeolylquinic acids, respectively [61]. 3-*p*-coumaroyl-5-caffeoylquinic acid (**45**) was deduced from the base ion at *m*/*z* 163.039 [*p*-coumaric acid−H]^−^, along with relevant ions at *m*/*z* 337.093 [M−H−caffeoyl]^−^ and *m*/*z* 119.049 [*p*-coumaric acid−H−CO_2_]^−^. The base peak at *m*/*z* 173.045 indicated substitution at position four of the quinic acid (**49**, **50**, **51**). Compounds **50** and **51** were assigned to 4-caffeoyl-5-*p*-coumaroylquinic acid and 4-feruloyl-5-caffeoylquinic acid due to the relative abundance of the ions at *m*/*z* 353.088 [M−H−*p*-coumaroyl]^−^ and 367.103 [M−H−caffeoyl]^−^, respectively.

##### Flavonoids

Significant for the aglycone annotation were the retro-Diels–Alder (RDA) rearrangements, supported by neutral losses of CO (−28 Da), CO_2_ (−44 Da), CH_2_O (−30 Da), and H_2_O (−18 Da) [61,62]. Neutral mass losses of 162, 146, and 176 Da were consistent with the removal of hexose, deoxyhexose, and glucuronic acid, respectively.

Two C-hexosides (**55** and **56**) were deduced from the losses of 90 and 120 Da resulting from the hexose cross-ring cleavages ^0.2^Hex and ^0.3^Hex and prominent ion fragments [M−H−2 × 120]^−^ at *m*/*z* 355.083 and 353.067, respectively, suggesting two hexose units with a C-glycosidic bond [63]. Accordingly, they were assigned as naringenin diC-hexoside (**55**) and apigenin diC-hexoside (**56**).

Regarding compound **60**, the neutral loss of 308 Da corresponded to the presence of rutinose. Additionally, the RDA ions at *m*/*z* 178.998 [^1,2^A−H]^−^, 151.002 [^1,3^A]^−^, 121.028 [^1,2^B]^−^, and 107.012 [^0,4^A]^−^ evidenced the aglycone quercetin. Thus, **60** was identified as rutin. **61** ([M−H]^−^ at *m*/*z* 477.0669) shared the same aglycone and afforded a prominent ion at *m*/*z* 301.034 [M−H−HexA]^−^, suggesting quercetin *O*-hexuronide.

Compounds **64** and **72** shared the same [M−H]^−^ at *m*/*z* 461.0736 and were tentatively identified as luteolin *O*-hexuronide and its isomer, based on the fragment ions at *m*/*z* 285.0405 [M−H−HexA]^−^ and the RDA ions at *m*/*z* 151.002 (^1,3^A^−^), 133.025 (^1,3^B^−^), and 107.012 (^0,4^A^−^).

Compound **83** yielded relevant fragment ions at *m*/*z* 447.0908 and 285.0406, resulting from caffeoyl and hexosyl moiety losses. In addition, the fragment ions at *m*/*z* 179.034 [(caffeic acid−H)]^−^, 161.023 [(caffeic acid−H)−H_2_O]^−^, and 135.044 [(caffeic acid−H)−CO_2_]^−^ corroborated luteolin *O*-caffeoylhexoside.

Compound **95** afforded fragment ions consistent with the consecutive losses of methoxy groups at *m*/*z* 344.054 [M−H−•CH_3_]^−^, 329.031 [M−H−2•CH_3_]^−^, and 314.006 [M−H−3•CH_3_]^−^. The ion at *m*/*z* 178.997 (^1,2^A^−^− •CH_3_) suggested a methoxy group at position six. Supported by the losses at *m*/*z* 148.015 [^1,3^B−•CH_3_-CH_2_]^−^ and 163.039 (^1,3^B^−^−CH_2_), it was assigned as quercetagetin-3,6,3′(4′)-trimethyl ether.

Luteolin 7-*O*-glucoside (**65**), isorhamnetin 3-*O*-glucoside (**73**), apigenin 7-*O*-glucoside (**74**), luteolin (**84**), quercetin (**85**), apigenin (**90**), kaempferol (**91**), isorhamnetin (**92**), and jaceosidin (**94**) were unambiguously identified by comparison with the fragmentation fingerprints of the reference standards.

### 2.2. Activity Cage

**Figure 2:**
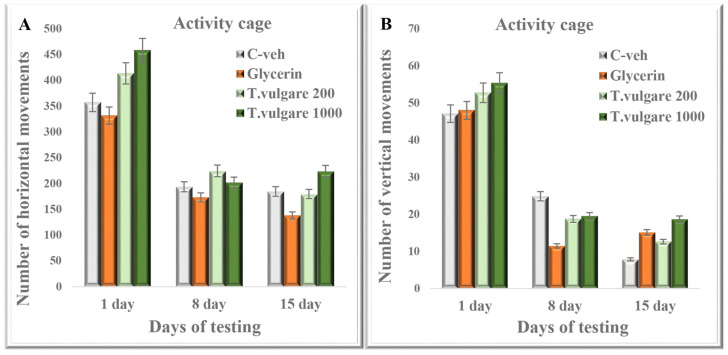
Effect of chronic treatment with *T. vulgare* alcohol extract on (**A**) the number of horizontal movements and (**B**) the number of vertical movements in the activity cage. Data are presented as means ± S.E.M. (n = 10 per group). One-way-ANOVA, followed by Tukey’s post hoc test showed no statistical significance between the experimental groups (*p* > 0.05).

The post hoc test did not show any statistical significance between the four experimental groups in the number of horizontal movements during all days of testing (Figure 2). Moreover, no difference in the number of vertical movements was observed between the control groups and the animals treated with different doses of *T. vulgare* extract (Figure 2).

### 2.3. Avoidances—Learning and Memory Sessions in a Shuttle Box

**Figure 3:**
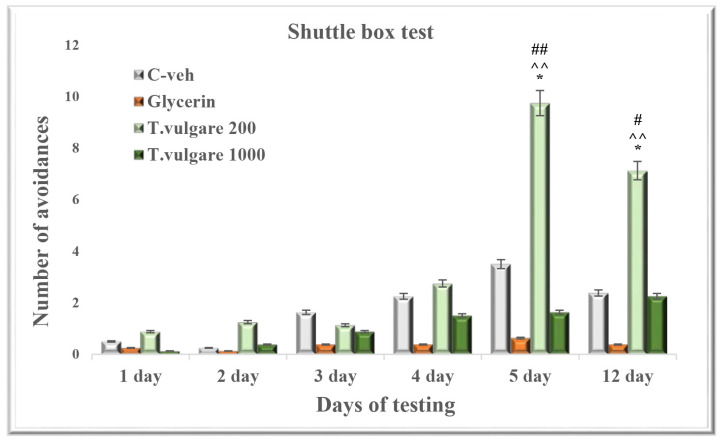
Effect of chronic treatment with *T. vulgare* alcohol extract on the number of avoidances during learning and memory tests in the Shuttle box. Data are presented as means ± S.E.M. (n = 10 per group). One-way-ANOVA followed by Tukey’s post hoc test showed: * *p* < 0.05 *T. vulgare* 200 compared to the C-veh group; ^^^^ *p* < 0.01 *T. vulgare* 200 compared to the glycerin group; ^#^ *p* < 0.05, ^##^ *p* < 0.01 *T. vulgare* 200 compared to the *T. vulgare* 1000 group.

The animals treated with the low dose of 200 mg/kg *T. vulgare* alcohol extract increased the number of avoidances during all days of the learning session, but the difference was significant only on day 5 in comparison with the C-veh (*p* < 0.05) and glycerin (*p* < 0.01) groups. Moreover, the *T. vulgare* 200 group also showed a significant increase in the number of conditioned stimulus responses compared to the animals treated with high dose of the alcohol extract on day 5 (*p* < 0.01) of the learning session (Figure 3).

During the retention test, the group treated with 200 mg/kg *T. vulgare* had significantly higher number of avoidances in comparison with the control animals (*T. vulgare* 200 vs. C-veh, *p* < 0.05; *T. vulgare* 200 vs. glycerin group, *p* < 0.01) and with the animals treated with 1000 mg/kg *T. vulgare* (*p* < 0.05) (Figure 3).

### 2.4. Escapes—Learning and Memory Sessions in a Shuttle Box

**Figure 4:**
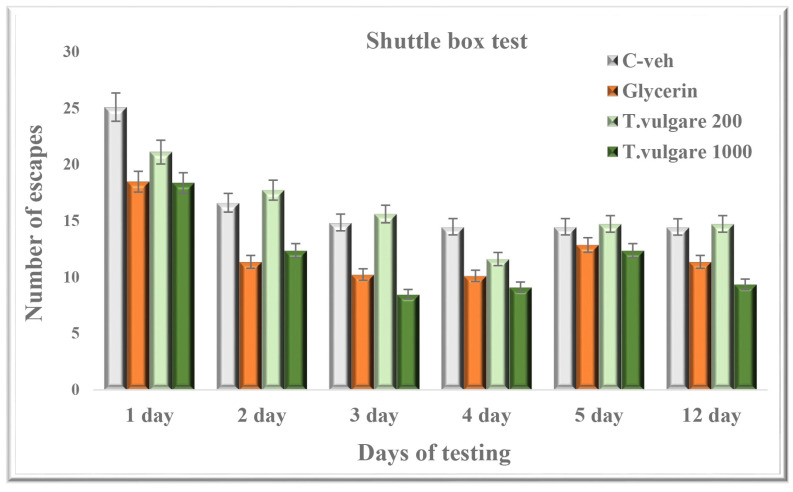
Effect of chronic treatment with *T. vulgare* alcohol extract on the number of passive avoidances during learning and memory tests in the shuttle box. Data are presented as means ± S.E.M. (n = 10 per group). One-way-ANOVA followed by the Games–Howell post hoc test showed no statistical significance between the experimental groups (*p* > 0.05).

The post hoc test did not reveal any significant changes between the four experimental groups on the number of escapes in the learning session or memory retention test (Figure 4).

### 2.5. Intertrial Crossings—Learning and Memory Sessions in the Shuttle Box

**Figure 5:**
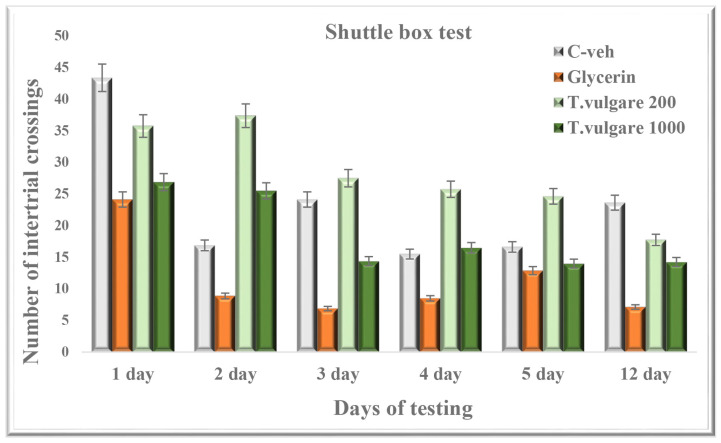
Effect of chronic treatment with *T. vulgare* alcohol extract on the number of intertrial crossings during learning and memory test in the shuttle box. Data are presented as means ± S.E.M. (n = 10 per group). One-way-ANOVA followed by the Games–Howell post hoc test showed no statistical significance between all experimental groups (*p* > 0.05).

No difference was found between the number of intertrial crossings of the experimental groups in comparison with the control animals during the learning and memory retention tests (Figure 5).

### 2.6. Latency of Reaction—Learning and Memory Sessions in the Step-Down Test

**Figure 6:**
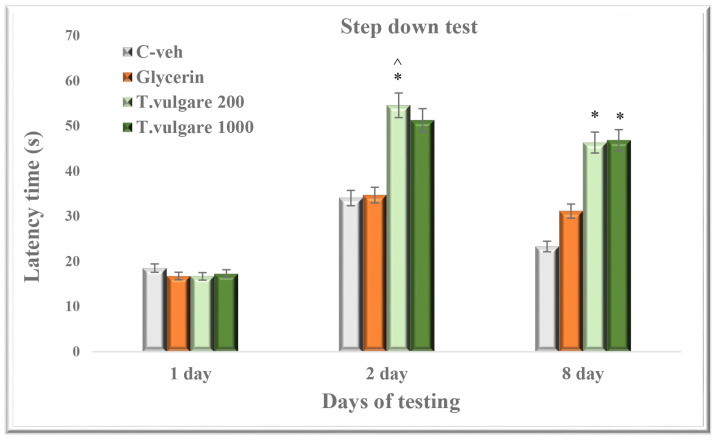
Effect of chronic treatment with *T. vulgare* alcohol extract on latency during learning and memory in the passive avoidance step-down test. Data are presented as means ± S.E.M. (n = 10 per group). One-way-ANOVA followed by Games–Howell post hoc test showed: * *p* < 0.05 *T. vulgare* 200 compared to the C-veh group; * *p* < 0.05 *T. vulgare* 1000 compared to the C-veh group; and ^^^ *p* < 0.01 *T. vulgare* 200 compared to the glycerin group.

In the passive avoidance step-down test, no change in the latency of reaction time was found between the four groups during the learning session on day 1 (Figure 6). The group treated with the low dose of 200 mg/kg *T. vulgare* showed increased latency during the short-term memory retention test compared to the control animals (*p* < 0.05). During the same session, the group treated with 1000 mg/kg alcohol extract showed only a strong tendency for longer latency in comparison with the C-veh rats (*p* = 0.08).

During the long-term memory retention test on day 8, both groups treated with *T. vulgare* alcohol extract increased the time spent on the platform compared to the control animals (*p* < 0.05 for both) (Figure 6).

### 2.7. Spatial Memory Assessed in the Y-Maze Test

**Figure 7:**
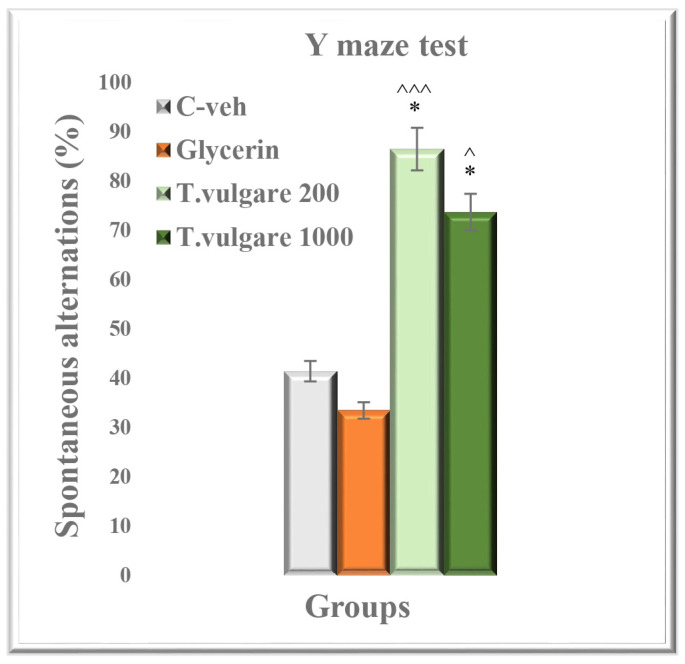
Effect of chronic treatment with *T. vulgare* alcohol extract on spontaneous alternations in the Y-maze test. Data are presented as means ± S.E.M. (n = 10 per group). One-way-ANOVA followed by Tukey’s post hoc test showed: * *p* < 0.05 *T. vulgare* 200 compared to the C-veh group; * *p* < 0.05 *T. vulgare* 1000 compared to the C-veh group; ^^^ *p* < 0.05 *T. vulgare* 1000 compared to the glycerin group; and ^^^^^ *p* < 0.001 *T. vulgare* 200 compared to the glycerin group.

The post hoc test revealed a significantly higher percentage of spontaneous alternations of both groups treated with alcohol extract of *T. vulgare* when compared to the control animals: *T. vulgare* 200 vs. C-veh group, *p* < 0.05 and vs. glycerin treated animals, *p* = 0.001; and *T. vulgare* 1000 vs. C-veh group and glycerin treated animals, *p* < 0.05, respectively (Figure 7).

### 2.8. Recognition Memory Assessed in the Object Recognition Test

**Figure 8:**
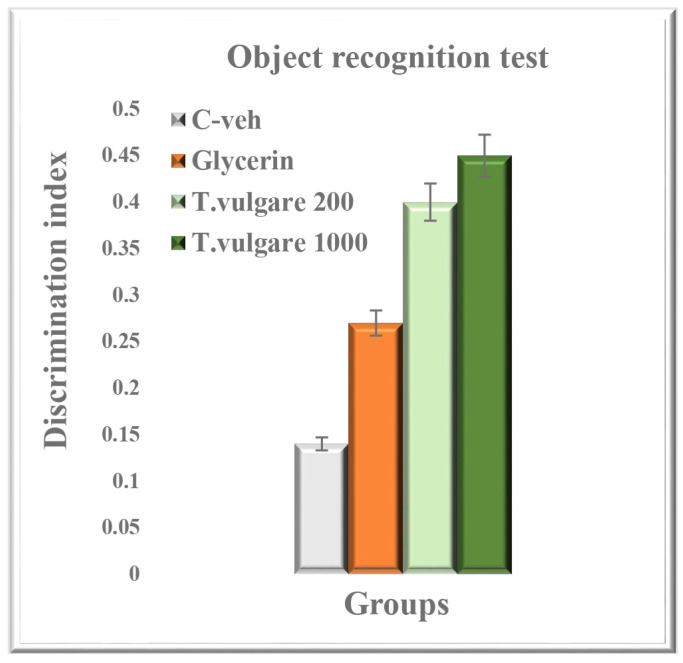
Effect of chronic treatment with *T. vulgare* alcohol extract on recognition memory in the object recognition test. Data are presented as means ± S.E.M. (n = 10 per group). One-way-ANOVA followed by Tukey’s post hoc test showed no statistical significance between experimental groups (*p* > 0.05).

Although both groups treated with *T. vulgare* alcohol extract had higher discrimination index (DI) values compared to the control groups, post hoc tests did not reveal any statistical significances (Figure 8).

### 2.9. Anxiety Index Assessed in the EPM Test

**Figure 9:**
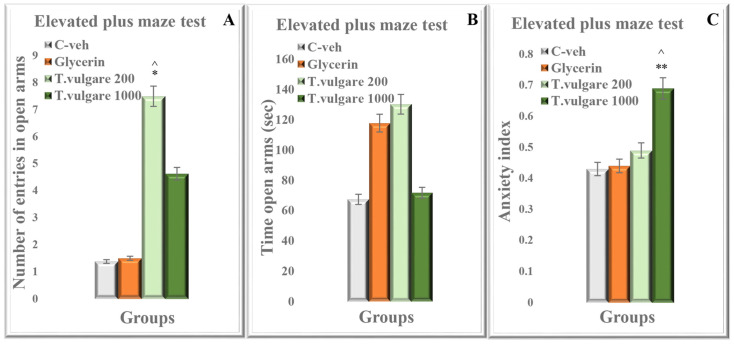
Effect of chronic treatment with *T. vulgare* alcohol extract. (**A**) Number of entries in the open arms of the EPM test. Data are presented as means ± S.E.M. (n = 10 per group). One-way-ANOVA followed by Tukey’s post hoc test showed * *p* < 0.05 *T. vulgare* 200 compared to the C-veh group and ^^^ *p* < 0.05 *T. vulgare* 200 compared to the glycerin group. (**B**) Time spent in the open arms of the EPM test. Data are presented as means ± S.E.M. (n = 10 per group). One-way-ANOVA followed by Tukey’s post hoc test showed no statistical significance between experimental groups (*p* > 0.05). (**C**) Anxiety index in the EPM test. Data are presented as means ± S.E.M. (n = 10 per group). One-way-ANOVA followed by Tukey’s post hoc test showed ** *p* < 0.01 *T. vulgare* 1000 compared to the C-veh group and ^^^ *p* < 0.05 *T. vulgare* 1000 compared to the glycerin group.

In the EPM test, one-way ANOVA demonstrated that the group treated with low-dose *T. vulgare* had a significantly higher number of entries in the open arms of the maze compared to both control groups C-veh and glycerin one (*p* < 0.05 for both) (Figure 9A). On the other hand, no difference between the experimental groups was observed in the time spent in the open arms (*p* > 0.05) (Figure 9B). A decreased anxiety index was established for the group treated with 1000 mg/kg *T. vulgare* in comparison with both groups C-veh (*p* < 0.01) and glycerin (*p* < 0.05) (Figure 9C). A strong tendency for a similar effect was observed by the animals treated with the low dose of the alcohol extract (*p* = 0.059).

### 2.10. Histological Results

**Figure 10:**
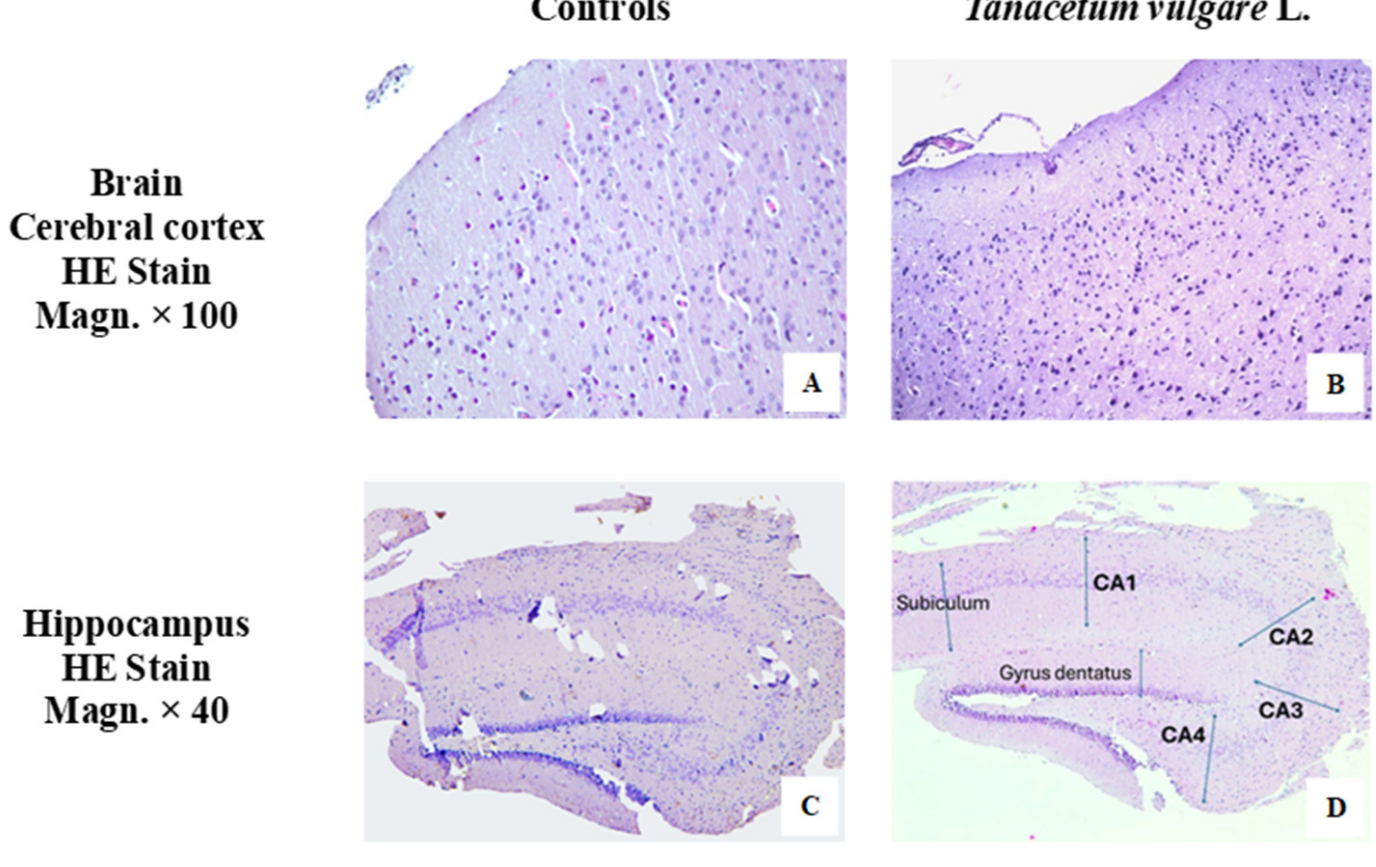
Cerebral cortex and hippocampus of animals from the control and treated groups, stained with hematoxylin and eosin (HE). Cerebral cortex with normal structure. A layered structure is preserved in the cerebral cortex in controls and treated animals. The superficial molecular layer has a reticular organization of nerve fibers with single neuroglial cells and neurons. In depth, granule and pyramidal neurons form the typical six-layer organization. Below the molecular layer is the well-formed external granular layer, represented by small pyramidal and granule neurons. Pyramidal neurons of the external and internal pyramidal layers have the typical arrangement with an elongated apical part directed to the surface of the cortex. (**A**) Cerebral cortex—controls. (**B**) Cerebral cortex—*T. vulgare* L. For the hippocampus with normal structure, from the bottom of the sulcus hippocampi to the cavity of the lateral ventricle, the hippocampal cortex is made up of the following layers: 1. stratum moleculare, 2. stratum lacunare, 3. stratum radiatum, 4. stratum pyramidale, and 5. stratum oriens. Only three of them (stratum moleculare, radiatum and pyramidale) are cellular—archeocortex. The main type of neurons of the hippocampus are large pyramidal cells located in the stratum pyramidale. Scattered stellate neurons with short axons are found in the stratum moleculare. Pyramidal and stellate cells are found in the stratum radiatum. Different regions differ in their neuronal structure. Small pyramidal neurons predominate in regions CA1 and CA2, large pyramidal neurons are located in CA3 and CA4, which projects into the recess of the gyrus dentatus, and is formed by small granule neurons. (**C**) Hippocampus—controls. (**D**) Hippocampus—*T. vulgare* L.

Figure 10 presents images of the histological examination of the cerebral cortex and hippocampus in controls and experimental groups (rats treated with different concentrations of *T. vulgare* L.: 200 mg/kg and 1000 mg/kg).

In the cerebral cortex sections, all neuronal layers from granule and pyramidal neurons and fibrous layers between them are clearly visible. The superficial molecular layer has a reticular organization of nerve fibers with single neuroglial cells and neurons. In depth, granule and pyramidal neurons form the typical six-layer organization (Figure 10A,B).

The cortex of the hippocampus consists of five layers, three of which are cellular. Due to differences in the construction of the layers, the hippocampus is divided transversely into four sectors: CA1, CA2, CA3 and CA4 (CA—from cornu Ammonis). No difference was found in the appearance of the hippocampal formation in the experimental animals and that in the controls in the thickness of the layers or in the neuronal composition (Figure 10C,D).

**Figure 11:**
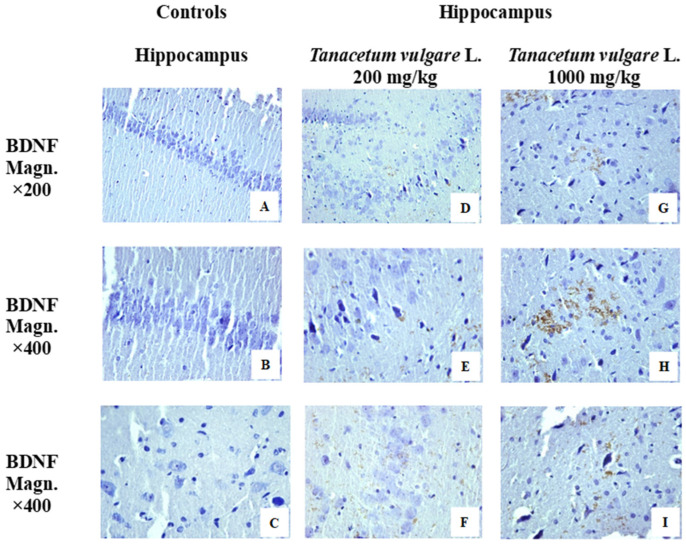
Immunohistochemical reactions for BDNF in hippocampus of animals from the control and treated groups. Hippocampus of controls: negative immunohistochemical expression in gyrus dentatus and CA3 region (**A**–**C**). Hippocampus of treated animals: positive reaction for BDNF. BDNF expression is concentrated in regions CA2 and CA3. Positive BDNF expression with homogeneous brown staining of the perikaryons of some large pyramidal neurons of the stratum pyramidale. A fine brown granulation is observed in the neurons of the stratum radiatum, as well as along the course of the neuronal processes in the fibrous layers ((**D**–**F**)—*T. vulgare* 200 mg/kg, (**G**–**I**)—*T. vulgare* 1000 mg/kg).

**Figure 12:**
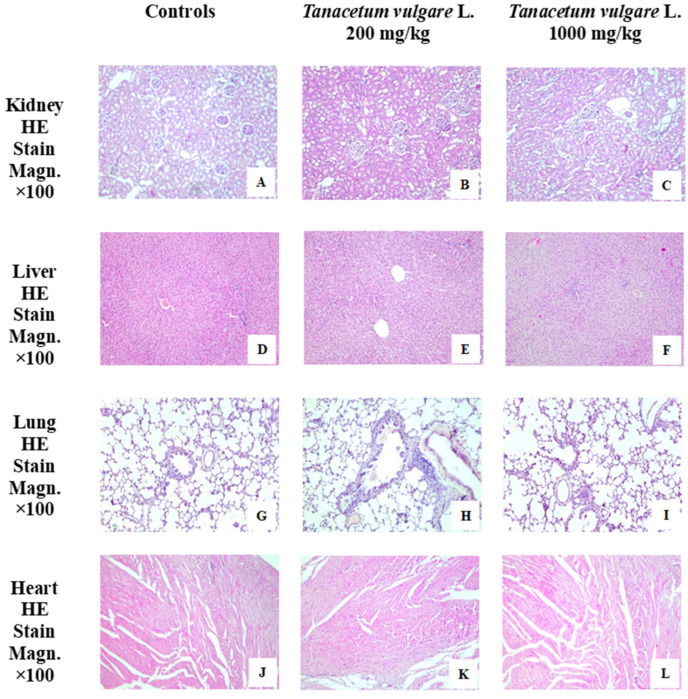
Kidney, liver, lung, and heart of animals from the control and treated groups, stained with hematoxylin and eosin (HE). For the kidney with normal structure, in the cortex, Malpighian bodies with preserved glomeruli and spaces of Baumann’s capsule without changes in the epithelium of the proximal and distal tubules, as well as in the wall of the blood vessels between them. The medulla is represented by straight portions of proximal, distal tubules and collecting tubules with cuboidal epithelium, as well as the loops of Henle with a thin descending limb and a thin ascending limb composed of simple squamous epithelia, and a thick ascending limb, composed of simple cuboidal epithelium. (**A**) Kidney—controls. (**B**) Kidney—*T. vulgare* (200 mg/kg). (**C**) Kidney—*T. vulgare* (1000 mg/kg). For the liver with normal structure, hepatocyte lamellae with typical radial organization around central vein. Some of the hepatocytes are binucleated, with a more intensely stained cytoplasm, a sign of functional activity. Between them is a labyrinth of sinusoids. Normal portal spaces with a scant amount of connective tissue. There is no inflammatory reaction or fibrosis in the area of the triads, represented by interlobular artery, vein and bile duct with cuboidal epithelium. (**D**) Liver—controls. (**E**) Liver—*T. vulgare* (200 mg/kg). (**F**) Liver—*T. vulgare* (1000 mg/kg). For lung with normal structure, sections of bronchi of different caliber with preserved ciliated epithelium and blood vessels from the functional circulation in the neighborhood. Terminal bronchioles with a normal lumen covered with simple cuboidal ciliated epithelium and well-defined smooth muscle cells. No inflammatory, necrotic, or fibrotic changes in the respiratory bronchioles and acini. (**G**) Lung—controls. (**H**) Lung—*T. vulgare* (200 mg/kg). (**I**) Lung—*T. vulgare* (1000 mg/kg). For heart with normal structure, myocardium of the heart with dense layers of cardiomyocytes in different directions. In the loose connective tissue between them, blood vessels with normal wall thickness and preserved endothelium. The cardiomyocytes are connected in a functional syncytium. They are supplied with blood by a well-developed capillary network between them. (**J**) Heart—controls. (**K**) Heart—*T. vulgare* (200 mg/kg). (**L**) Heart—*T. vulgare* (1000 mg/kg).

The immunohistochemical analysis for BDNF in hippocampal sections is demonstrated in Figure 11. In the controls, the BDNF reaction was negative (Figure 11A–C). BDNF expression was absent in both neuronal and fibrous layers of the hippocampus. The expression of BDNF in the hippocampus of the experimental groups, rats treated with different concentrations of *T. vulgare* L., is positive. It is concentrated in regions CA2 and CA3. BDNF expression in the gyrus dentatus is in the stratum polymorphe and stratum moleculare (Figure 11D–F—*T. vulgare* 200 mg/kg) (Figure 12G–I—*T. vulgare* 1000 mg/kg).

Figure 12 presents images of the histological examination of kidney, liver, lung, and heart in the first group, controls, and rats treated with different concentrations of *T. vulgare* extract: second group, *T. vulgare* (200 mg/kg), and third group, *T. vulgare* (1000 mg/kg). They show normal structure of the organs in both groups of animals.

In the kidney sections, normal structure of the cortical labyrinth and medulla is observed. Malpighian corpuscles show visible vascular poles. Between them, proximal and distal convoluted tubules with well-stained cytoplasm of the cuboidal epithelium are densely located (Figure 12A–C).

The liver slides showed no difference between animals from the control groups and the treated groups. The classic hepatic lobule with a normal structure and hexagonal shape is observed. Hepatocyte lamellae have a radial direction around the central vein. Triads of interlobular artery, vein and bile duct and a scant amount of connective tissue are located in the portal space (Figure 12D–F).

In the lung of control and treated rats, we observed normal lung parenchyma. The transverse sections show bronchi of different diameters with preserved ciliated epithelium, blood vessels, respiratory bronchioles, and alveoli between them (Figure 12G–I).

The heart wall from the the control and experimental groups of animals showed no deviations from the normal structure. We can see layers of cardiomyocytes with a clearly visible transverse striation, located in different directions and blood vessels between them. Branches of the coronary atria are located among the small number of adipocytes in the subepicardium (Figure 12J–L).

## 3. Discussion

In the current study, we investigated the effect of *T. vulgare* alcohol extract on various cognitive domains based on multiple behavioral tests. Some of them included negative reinforcement and examined active and passive learning and memory, while recognition and spatial memory were based on tests that do not require any external motivation, punishment, or reward. The findings from the present study suggest that *T. vulgare* alcohol extract can positively influence only some of the cognitive domains.

It was established that the low dose of 200 mg/kg of the extract exhibited a greater effect than the higher dose of *T. vulgare* in some of the tests. In the active avoidance task with negative reinforcement, only *T. vulgare* at 200 mg/kg improved aversive learning and long-term memory retention. Moreover, both doses improved the formation of short- and long-term memory traces in the inhibitory avoidance task without affecting passive learning. Many brain structures play an important role in the two-way active avoidance task in rodents, such as the hippocampus, frontal cortex, and amygdala, sensory, visual, and limbic cortex participate. This is mainly due to the complexity of this test, as it combines both instrumental avoidance conditioning and classic fear conditioning [64]. In addition, this test examines a non-declarative (implicit) memory, while declarative (explicit) memory is investigated by passive avoidance tests where the hippocampus plays a crucial role in the acquisition and consolidation of new information [65]. In the current study, we found that the alcohol extract of *T. vulgare* improved hippocampus-associated spatial memory as a result of an increased number of spontaneous alternations assessed in the Y-maze test. This test is based on the natural exploratory behavior of rodents [66]. Despite the increased discrimination index by both doses of the plant’s extract, no significant effect was observed on object recognition memory. This test is based on spontaneous novelty preference and it is associated with different brain structures, mainly the dorsal hippocampus [67].

Additionally, we investigated the locomotor activity of both doses *T. vulgare* in two different apparatuses—the activity cage and shuttle box. We found no change in the number of interatrial crossings and horizontal and vertical movements, which shows a lack of hyperactivity in the rodents and no risk of pseudo-positive results in the cognitive-assessment tests.

Moreover, in the present study, we explored the anxiolytic effect of the alcohol extract of *T. vulgare* and we found that the low dose of the extract increased the number of entries in the aversive area of the maze, while the high dose of 1000 mg/kg decreased significantly the level of anxiety. Considering these results, we can propose that the observed anxiolytic effect of the extract could be one of the mechanisms leading to better cognitive performance, especially in the tasks with negative reinforcements.

As a possible mechanism of cognition-improving outcomes of *T. vulgare*, we investigated the effect of the alcohol extract on brain-derived neurotrophic factor (BDNF) in the hippocampus. We found that both doses of the extract significantly increased the expression of BDNF in this brain structure, especially in the CA2 and CA3 subregions and in the dentate gyrus as well. It is well known that BDNF plays a crucial role in synaptic plasticity, promoting neurotransmission and regulating receptor sensitivity [68]. Moreover, hippocampal BDNF is essential for the induction and maintenance of stable long-term potentiation (LTP), which is regarded as a cellular basis for learning and memory [69]. As a key regulator of hippocampal neuronal plasticity and protein synthesis, increased levels of BDNF are associated with better cognitive performance. This is in line with our current results, and we suggest that increased neurotrophic factor expression is one of the important mechanisms underlying the cognition-enhancing effects of *T. vulgare* extract in hippocampal-dependent cognitive tasks.

Although *T. vulgare* has been widely used in traditional phytomedicine as an anti-inflammatory, anti-helminth, and wound healing agent, little is known about its neuromodulatory properties. There are few studies that have investigated the neuroprotective effect of herbal combinations containing *T. vulgare*, *Rosa canina* and *Urtica dioica* in different models of impaired cognitive functions. Ghanbari et al. demonstrated anti-aging properties with a reduction in serum pro-inflammatory cytokines (IL-α, IL-1β, and IL-6) and pronounced anti-oxidative stress potential [70], which aligns with our previous work, where we found that *T. vulgare* essential oil exerted strong antioxidant activity [71]. In accordance with our current results, another study has revealed in a rat model of sporadic Alzheimer’s disease that the extract of the three herbs had pronounced anti-dementia properties and led to improved spatial learning and memory. As possible mechanisms of these effects, the authors suggested increased hippocampal synaptophysin expression and decreased *Psen1* expression [72]. Additionally, literature data have demonstrated that the herb combination protects the brain from ischemic cerebral injury and improves motor function as a result of decreased oxidative stress and neuroinflammation leading to better cerebral blood flow [73].

Plant phenolic compounds have been reported to exert a plethora of biological activities such as antibacterial, antioxidant, anticancer, cardioprotective, anti-inflammatory, etc. [74,75,76,77]. Notably, they exhibit positive effects on the cognitive functions through various pathways [78,79,80].

The extract was found to contain significant levels of chlorogenic acid (6.886 mg/g). It has been implicated in exhibiting neuroprotective effects, mainly due to its antioxidant properties, since the brain is especially vulnerable to damage caused by oxidative stress [81]. Kim et al. reported protective effects of chlorogenic acid against oxidative neuronal death inhibiting apoptotic nuclear condensation induced by H_2_O_2_ [82]_._ Cho et al. also found that chlorogenic acid reduces H_2_O_2_-induced PC12 neuronal cell death [83]. Additionally, according to Kwon et al., it exerts neuroprotective effects on scopolamine-induced amnesia in mice and improves short-term or working memory through inhibition of acetylcholinesterase and malondialdehyde in the hippocampus and frontal cortex [84]. A beneficial effect of chlorogenic acid on spatial memory in animals has also been demonstrated after transient global ischemia [85] and in a model of age-related cognition decline (SAMP8 mice) [86]. Moreover, in a randomized, double-blind, placebo-controlled trial, Saitou et al. established improvement in some cognitive functions in middle-aged and elderly individuals after a 16-week intake of chlorogenic acids (caffeoylquinic acids, feruloylquinic acids, and dicaffeoylquinic acids) [87]. A recent study found a preventive effect of chlorogenic acid on BDNF depletion and alleviation of astrogliosis and microgliosis in the prefrontal cortex and hippocampus in a model of sporadic Alzheimer’s disease [88]. We suggest that the effects of the extract could be associated with the chlorogenic acid content. However, the synergism between the main compounds found in the extract should be considered as well.

Rutin (quercetin-3-O-rutinoside), another predominant constituent of the extract, for instance, has also demonstrated neuroprotective effects in vitro and in vivo in several models of neurodegenerative diseases that could be linked to its antioxidant, anti-apoptotic, and anti-inflammatory properties, as well as activation of BDNF and the MAPK cascade [89,90,91]. Asghariana et al. observed a beneficial effect of rutin as the major compound in hydroalcoholic extract of *Ruta graveolens* on memory and learning performance in rats due to potent antioxidant activity and decreased levels of serum and brain MDA [92]. In a mouse model of Alzheimer’s disease, Sun et al. reported inhibitory effect of rutin on tau aggregation and tau oligomer-induced cytotoxicity, as well as lowered neuroinflammation, leading to a significant improvement in cognition [93]. Moreover, it improved the spatial working memory of rats exposed to cadmium trough cholinesterase inhibition and enhanced nitric oxide and antioxidant status [94].

Furthermore, no histopathological changes in the internal organs (kidney, liver, lung, heart) of the treated animals were observed in the herein investigation. These findings, along with previous in vivo studies on the toxicity of the plant (essential oil [71], aqueous extract [95]), represent a crucial key point for future research regarding the application perspectives of tansy extracts.

## 4. Materials and Methods

### 4.1. Plant Material

The inflorescences of wild-grown *T. vulgare* L. were collected in the phase of full flowering from the region of Tsigov Chark, Batak (western Rhodope Mountains, Bulgaria). The plant was authenticated by Associate Professor Niko Benbassat based on Flora of the Republic of Bulgaria [96]. A voucher specimen (063584) was deposited in the Herbarium of the University of Agriculture, Plovdiv, Bulgaria. Thereafter, the plant material was dried at room temperature (20–25 °C).

### 4.2. Extract Preparation

The powdered plant material was extracted with 70% ethanol using the Soxhlet method (hydromodule 1:15). The extraction was carried out for 6 h. The extract was filtered and concentrated using a rotary evaporator. The dry residue of the extract was determined according to the *European Pharmacopoeia* [97].

### 4.3. Chemical Profile

The HPLC-UV analyses were carried out by a method previously reported in many other studies [56,57,58,59]. The UHPLC-HRMS analyses were performed as described by Ak et al. [98].

### 4.4. Animals

In the current study, we used forty mature male Wistar rats with body weight of 140–160 g. They were obtained from the Animal Center of the Medical University, Plovdiv. The animals were housed in plastic cages. Food and drinking water were allowed ad libitum. The rats were maintained under standard laboratory conditions (12/12 h dark/light cycle, 21–25 °C temperature, 55 ± 5% humidity). All procedures were in accordance with the Bulgarian Food Safety Agency (permission 238/2019, valid until 11 September 2024) and were formally approved by the Ethics Committee on Human and Animal Experimentation of the Medical University of Plovdiv. This study was performed in strict accordance with the guidelines of the European Community Council directive 86/609/EEC.

The rats were randomly divided into 4 groups as follows:

1st group, C-veh, treated with saline 1 mL p.os;
2nd group, glycerin group, treated with glycerine 1mL p. os;
3rd group, *T. vulgare* 200, treated with 200 mg/kg extract of *T. vulgare* p.os;
4th group, *T. vulgare* 1000, treated with 1000 mg/kg extract of *T. vulgare* p.os.

All animals were treated daily during the whole testing period. The plant extract and the vehicles were administered 30 min before each test.

### 4.5. Behavioral Tests

**Figure 13:**
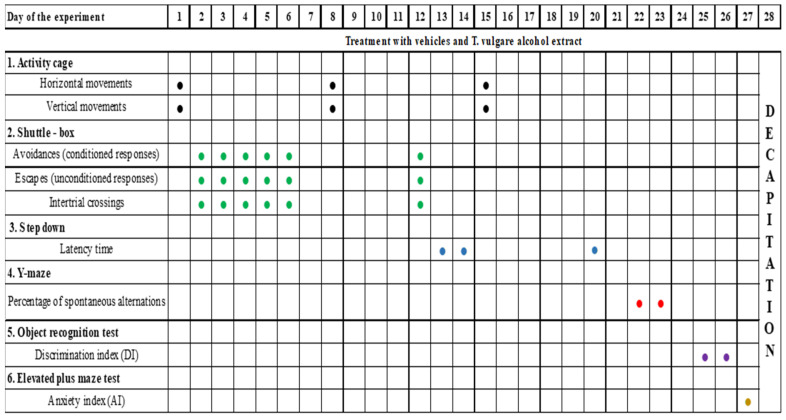
Design of the experimental protocol.

Figure 13 represents a timeline of all behavioral tests conducted.

#### 4.5.1. Activity Cage Test

The activity cage (Biological Research Apparatus, UgoBasile, Italy) was a clear plastic box 40 cm square with 40 cm-high walls. A printer automatically recorded the number of movements in the two categories, horizontal and vertical, captured by the infrared sensor array located on either side of the cage. The animals were individually tracked for 3 min in each session, which was conducted under identical conditions. This testing was performed on days 1, 8, and 15 from the beginning of the experiment, measuring spontaneous locomotor activity.

#### 4.5.2. Active Avoidance Test Shuttle Box

The active avoidance test was performed in a shuttle box (Automatic Reflex Conditioner, UgoBasile, Italy). The box had two chambers separated by a constantly opened gate allowing the subject to pass freely. The floor consisted of stainless steel rods spaced 1 cm apart through which electrical shocks were delivered. The wall had sensors that monitored the movements of the rats between chambers. Learning sessions were held for 5 consecutive days and consisted of thirty trials each. On day 12, the memory retention test was performed. During each task session, the rat was given conditioned stimuli by using a light and buzzer (670 Hz, 70 dB, 6 s) and an unconditioned stimulus—the electric foot shock (0.4 mA, 3 s). The interval between conditioned and unconditioned stimuli was 12 s. The following behavioral parameters were recorded: number of avoidances (movements through the gate while conditioned stimuli are on), number of escapes (moving to the other chamber upon receiving foot-shock) and number of intertrial crossings (movements through the gate after foot-shock cessation and before the next conditioned stimuli).

#### 4.5.3. Passive Avoidance Test with Negative Reinforcement Step-Down

The step-down passive avoidance test was performed in a compartment cage (UgoBasile, Italy) with a 7 cm plastic platform elevated above the floor grid. Two learning sessions were carried out on day 1. The short-term memory retention test was performed on day 2, and the long memory retention test on day 8. Each training session lasted 60 s. If the animal stepped off the platform onto the electrified grid, it was given an electrical stimulation of duration 10 s and intensity 0.4 mA. The latency of reactions (the rat remaining on the platform for more than 60 s) was taken as a criterion for learning and retention.

#### 4.5.4. Y-Maze Test

The Y-maze test is based on rodents’ natural exploratory instincts and is applied to assess spatial working memory [99]. In the experiment, we used a Y maze made of black Plexiglas. The three arms of the maze are interconnected at an angle of 120°. Each arm was randomly marked as A, B, and C. The experiment was conducted on two consecutive days: training session and behavioral testing. The animal was placed at the same end of one arm and allowed to move freely through the maze during a 5 min session. The number of alternations was calculated based on the sequences of arm entries. An alternation was defined as successive entries into 3 arms on an overlapping triple set—for example, ABC, CBA, CAB, BCA, etc. The percentage of spontaneous alternations was defined with the formula: SA (%) = (number of alternations)/(total number of entries − 2) × 100.

#### 4.5.5. Object Recognition Test

The object recognition test (ORT) consisting of an exploration session and a test session was carried out in two consecutive days. The object recognition test was performed in an open Plexiglas box (60 × 60 × 40cm), with objects made of plastic as previously described [100,101]. In brief, during the exploration session, each rat was allowed to explore two identical objects (A1 and A2) for 5 min. In the test session, one of the familiar objects was replaced with a novel one (B) and rats were allowed to investigate them for 5 min. The exploration time (seconds) for each object in the test session was recorded. The discrimination index (DI) was defined by the difference in exploration time between the novel and familiar objects, divided by the total time spent exploring these two objects in the discrimination phase: (TB − TA)/(TB + TA).

#### 4.5.6. Elevated Plus Maze Test

The apparatus consisted of two open arms (50 × 10 cm^2^), two enclosed arms (50 × 10 × 50 cm^3^) and a central platform (10 × 10 cm^2^) elevated 50 cm above the floor. The rats were placed on the central square of the maze facing an open arm and were allowed 5 min to freely explore the maze. The calculated measures were: (1) number of entries in open arms; (2) number of entries in enclosed arms; (3) time (seconds) spent in the open arms; and (4) anxiety index (AI) = 1 − [(open arms time/total time) + (number of entries in open arms/total number of entries)/2] [101].

### 4.6. Histological Analysis

One day after the last behavioral test, all animals were decapitated. The samples for histological and immunohistochemical studies from the brain and internal organs were taken after euthanasia of the rats from the tested groups at the end of the experiment.

Brains, kidneys, livers, lungs, and hearts of animals from the control and treated groups were fixed in 10% neutral formalin and embedded in paraffin. Paraffin sections with a thickness of 5 µm were stained with hematoxylin–eosin for the histological study. This staining allows common estimation of the organic structure and pathological alteration. The acquired samples were observed using an Olympus light microscope and microphotographic images were supplied via the microscope camera.

### 4.7. Immunohistochemistry

The immunohistochemical reaction was performed using the ABC method with a rabbit ABC staining system (Santa Cruz Biotechnology, Dallas, TX, USA) and primary antibody: anti-BDNF antibody (mouse brain-derived neurotrophic factor monoclonal antibody, MBS21105750, MyBioSurce, San Diego, CA, USA). Paraffin sections with a thickness of 5 µm were deparaffinized and incubated for 30 min in 2% H_2_O_2_ methanol to inactivate endogenous peroxidase. The primary anti-BDNF antibody was diluted in PBS at a ratio of 1:200. The sections were incubated at 4 °C for 12 h in a humid chamber with the biotinylated secondary antibody for 30 min and ABC complex for 15 min, then visualized with DAB chromogen. The deparaffinized and Vecta mount sections were examined and photographed by a Leica DM 3000 microsystem (Wetzlar, Germany).

### 4.8. Statistical Analysis

All statistical analysis were performed with the IBM SPSS^®^ (version 19.0) statistical package. Data are presented as means ± SEM. The results were analyzed using parametric tests for normally distributed data and assessed by the Kolmogorov–Smirnov test. The results of all behavioral tests were analyzed using one-way ANOVA. When the F ratio was significant, the between-group differences were assessed by the Tukey post hoc test for justification. When the variances were significantly different, depending on the homogeneity of the dispersions (found by using Levene’s test), the Games–Howell post hoc test was applied. Differences with *p* < 0.05 were considered statistically significant.

## 5. Conclusions

The findings from the present study suggest that *T. vulgare* alcohol extract can positively influence some domains of cognitive function. Moreover, the intake of the higher dose of the alcohol extract of *T. vulgare* (1000 mg/kg) was associated with significantly decreased levels of anxiety. We suggest that increased hippocampal neurotrophic factor expression could be one of the important mechanisms underlying the cognition-enhancing effects of *T. vulgare* extract. We consider that *T. vulgare* alcohol extract has promising therapeutic potential for the management of neurodegenerative/cognitive disorders. However, more studies are needed in this direction.

## Figures and Tables

**Table 1:** HPLC analysis of phenolic acids and flavonoids in *Tanacetum vulgare* extract.

Compound	Concentration, mg/g
Chlorogenic acid	6.886
*p*-Coumaric acid	0.041
Ferulic acid	3.585
Salicylic acid	7.519
Rutin	4.311
Quercetin	0.623
Kaempferol	2.265

## Data Availability

Data are contained within the article and Supplementary Materials.

## Supplementary Materials

The following supporting information can be downloaded at https://www.mdpi.com/article/10.3390/molecules29235723/s1. Figure S1: MS/MS spectra of hydroxybenzoic, hydroxycinnamic acids, and their derivatives (for the compound numbers, see Table A1); Figure S2: MS/MS spectra of acylquinic acids (for the compound numbers, see Table A1); Figure S3: MS/MS spectra of flavonoids (for the compound numbers, see Table A1).

## Appendix A

**Table A1:** Secondary metabolites in the *Tanacetum vulgare* extract.

№	Identified/Tentatively Annotated Compound	Molecular Formula	Exact Mass [M−H]^−^	Fragmentation Pattern in (-) ESI-MS/MS	t_R_ (min)	Δ ppm
Hydroxybenzoic, Hydroxycinnamic, and Phenylethanoid Glycosides						
1.	Gallic acid *	C_7_H_6_O_5_	169.0143	169.0132 (34.4), 125.0230 (100), 107.0124 (0.32), 97.0279 (3.04)	1.67	−6.015
2.	Protocatechuic acid-*O*-hexoside	C_13_H_16_O_9_	315.0727	315.0724 (100), 153.0182 (28), 152.0104 (56.4), 123.0074 (2.06), 108.0202 (81.93)	2.39	0.935
3.	Protocatechuic acid-*O*-hexoside isomer	C_13_H_16_O_9_	315.0727	315.0725 (100), 153.0182 (57.12), 109.0280 (79.33)	2.80	1.094
4.	Protocatechuic acid *	C_7_H_6_O_4_	153.0182	109.0280 (100), 91.0174 (1.5), 81.0330 (1.36)	2.90	−7.594
5.	Syringic acid-*O*-hexoside	C_15_H_20_O_10_	359.0985	359.0988 (8.3), 239.0564 (1.0), 197.0448 (100), 182.0212 (19.9), 166.9976 (8.3), 153.0546 (15.7), 138.0310 (27.7), 123.0072 (39.99)	3.11	0.641
6.	Caffeic acid-*O*-hexoside	C_15_H_18_O_9_	341.0871	341.0880 (20.7), 281.0663 (0.9), 251.0562 (18.2), 221.0447 (0.6), 179.0341 (100), 161.0233 (43.3), 135.0437 (63.5), 133.0282 (3.8)	3.38	0.424
7.	Quinic acid	C_7_H_12_O_6_	191.0549	191.0550 (100), 173.0443 (1.9), 155.0336 (0.2), 191.0553 (100), 173.0444 (2.04), 127.0387 (3.92), 111.0437 (1.36), 93.0330 (5.68), 85.0279 (17.37)	4.11	−4.404
8.	Aesculetin-*O*-hexoside	C_15_H_15_O_9_	339.0724	339.0724 (22.24), 177.0184 (100), 149.0221 (0.77), 133.0282 (9.51), 105.0331 (3.24), 89.0382 (2.21)	3.56	0.609
9.	Caffeoylgluconic acid	C_15_H_18_O_10_	357.0827	357.0839 (12.5), 195.0503 (100), 179.0340 (47.7), 177.0396 (11.5), 135.0439 (45.5), 129.0177 (4.3), 87.6073 (7.9), 73.0279 (5.2)	3.60	3.277
10.	Hydroxybenzoic acid-*O*-hexoside	C_13_H_16_O_8_	299.0778	299.0778 (1.33), 137.0231 (100), 93.0331 (49.7)	3.60	1.837
11.	Caffeoyl-O-hexose	C_15_H_18_O_9_	341.0871	341.0880 (21.9), 281.0667 (91.3), 251.0560 (57.7), 221.0452 (45.6), 179.0342 (100), 161.0235 (65.4), 135.0438 (70.1), 133.0282 (26.9)	3.64	0.688
12.	Caffeoylgluconic acid isomer	C_15_H_18_O_10_	357.0827	357.0828 (35.8), 195.0503 (100), 179.0342 (5.1), 177.0398 (4.6), 135.0439 (8.0), 129.0180 (12.3), 87.0072 (3.9), 59.0122 (2.6)	3.84	0.280
13.	Caffeic acid-*O*-hexoside isomer	C_15_H_18_O_9_	341.0871	341.0878 (25.7), 281.0665 (9.4), 251.0561 (5.8), 221.0454 (6.0), 179.0340 (100), 161.0232 (6.9), 135.0438 (66.1)	3.95	0.336
14.	*p*-coumaric acid	C_9_H_8_O_3_	163.0392	In Full MS 163.0392	4.01	−5.198
15.	Gentisic acid *	C_7_H_6_O_4_	153.0181	153.0181 (76.17), 135.0075 (33.46), 109.0280 (100), 91.0175 (6.27)	4.36	−7.790
16.	Caffeic acid *	C_9_H_8_O_4_	179.0339	179.0341 (19.62), 135.0438 (100), 117.0331 (0.8), 107.0487 (1.33)	4.58	−4.480
17.	Ferulic acid	C_10_H_10_O_4_	193.0506	In Full MS 193.0501	5.53	−2.549
18.	Feruloyl-(gentisyl)-dipentoside	C_27_H_30_O_15_	593.1507	593.1519 (100), 417.1042 (41.2), 399.0931 (19.8), 285.0634 (0.6), 153.0157 (2.8), 152.0104 (59.2), 134.0360 (17.8), 193.0500 (7.4), 108.0203 (46.1)	7.30	1.124
19.	Caffeic acid *O*-(hydroxybensoyl)-hexoside	C_22_H_22_O_11_	461.1089	461.1098 (49.), 323.0776 (24.8), 299.0785 (1.1), 179.0342 (3.3), 161.0232 (22.8), 137.0231 (100), 135.0440 (6.3), 133.0284 (12.0), 93.0331 (5.3)	7.42	1.816
20.	Shikimic acid	C_7_H_10_O_5_	173.0455	173.0445 (100), 155.0339 (3.44), 137.0229 (2.2), 127.0388 (1.76), 111.0437 (11.81), 93.0330 (80.04)	4.10	−6.106
	Mono-, diacyl-, and triacylquinic acids					
21.	Neochlorogenic (3-caffeoylquinic) acid *	C_16_H_18_O_9_	353.0867	353.0875 (3.0), 191.0553 (100), 179.0337 (1.2), 173.0442 (0.5), 161.0238 (1.8), 135.0437 (45.3), 217.0387 (2.0), 111.0436 (1.0), 93.0332 (2.9), 85.0279 (9.0)	2.78	−0.808
22.	Chlorogenic (5-caffeoylquinic) acid *	C_16_H_18_O_9_	353.0874	353.0880 (34.4), 191.0553 (100), 179.0340 (58.2), 173.0444 (3.6), 161.0234 (4.1), 135.0439 (47.8), 127.0387 (1.8), 111.0435 (0.7), 93.0331 (4.5), 85.0280 (9.2)	3.15	0.495
23.	3-*p*-coumaroylquinic acid	C_16_H_18_O_8_	337.0928	337.0936 (7.6), 191.0553 (8.7), 173.0444 (3.9), 163.0390 (100), 135.0434 (1.0), 127.0390 (0.4), 119.0489 (24.5), 111.0437 (1.1), 93.0329 (2.6), 85.0277 (1.5)	3.84	2.104
24.	4-feruloylquinic acid	C_17_H_20_O_9_	367.1034	367.1035 (93.5), 193.0500 (9.7), 173.0446 (69.2), 134.0360 (1.1), 111.0437 (14.5), 93.0331 (100), 85.0281 (0.7)	5.62	−0.015
25.	Caffeoylquinic acid dimer	C_32_H_36_O_18_	707.1829	353.0880 (53.2), 191.0553 (100), 173.0444 (5.7), 161.0230 (2.3), 135.0437 (4.5), 127.0388 (3.4), 111.0435 (1.7), 93.0331 (6.0), 85.0279 (12.1)	4.12	0.994
26.	3-feruloylquinic acid	C_17_H_20_O_9_	367.1034	367.1035 (19.8), 193.0498 (100), 191.0555 (3.6), 173.0444 (4.1), 149.0596 (3.1), 134.0360 (52.9), 127.0387 (1.8), 111.0437 (1.1), 93.0331 (1.8)	4.26	−0.015
27.	3-hydroxy-dihydrocaffeoyl-5-caffeoylquinic acid	C_25_H_26_O_13_	533.1301	533.1302 (70.8), 371.0983 (17.0), 353.0878 (18.5), 335.0779 (4.9), 191.0553 (100), 179.0341 (43.7), 173.0446 (14.4), 161.0235 (7.6), 135.0438 (58.1), 111.0437 (1.4), 93.0330 (7.0), 85.0280 (6.5)	4.83	0.236
28.	3-caffeoyl-5-hydroxy-dihydrocaffeoylquinic acid	C_25_H_26_O_13_	533.1301	533.1309 (79.4), 371.1002 (14.7), 353.0883 (20.9), 335.0776 (5.1), 209.0295 (18.0), 191.0553 (100), 179.0341 (50.6), 173.0445 (16.5), 161.0233 (8.7), 135.0438 (54.9), 111.0435 (2.2), 93.0331 (7.7), 85.0280 (14.0)	4.92	1.493
29.	5-*p*-coumaroylquinic acid	C_16_H_18_O_8_	337.0928	337.0934 (8.0), 191.0553 (100), 173.0446 (6.0), 163.0389 (6.3), 145.0282 (0.6), 119.0487 (5.1), 111.0436 (1.7), 93.0331 (16.3), 85.0281 (4.4)	4.99	1.511
30.	1,3-dicaffeoylquinic acid	C_25_H_24_O_12_	515.1190	515.1192 (78.5), 353.0880 (32.0), 335.0777 (12.4), 191.0553 (100), 179.0341 (67.3), 161.0234 (9.8), 135.0439 (63.7), 127.0388 2.5), 111.0436 (3.3), 93.0331 (6.5), 85.0280 (8.4)	5.04	−0.581
31.	3-hydroxy-dihydrocaffeoyl-4-caffeoylquinic acid	C_25_H_26_O_13_	533.1301	533.1300 (100), 371.098 (78.5), 353.0878 (12.9), 335.0779 (18.7), 197.0444 (6.6), 191.0552 (22.7), 173.0446 (54.3), 161.0233 (26.3), 135.0438 (86.1), 111.0436 (4.8), 93.0331 (15.9), 85.0277 (3.4)	5.25	−0.120
32.	1-caffeoyl-3-hydroxy-dihydrocaffeoylquinic acid	C_25_H_26_O_13_	533.1301	533.1311 (15.5), 371.0986 (58.3), 353.0876 (4.2), 335.0786 (1.5), 197.0454 (1.6), 191.0554 (18.5), 179.0339 (7.0), 173.0446 (11.4), 161.0238 (2.2), 135.0439 (100), 93.0328 (2.4), 85.0276 (1.7)	5.32	1.943
33.	5-feruloylquinic acid	C_17_H_20_O_9_	367.1034	367.1035 (18.1), 193.0502 (3.9), 191.0553 (100), 173.0446 (9.0), 134.0359 (7.3), 111.0437 (2.8), 93.0331 (18.3), 85.0280 (4.6)	5.40	0.176
34.	5-*p*-coumaroylquinic acid isomer	C_16_H_18_O_8_	337.0929	337.0933 (7.1), 191.0553 (100), 173.0445 (2.4), 163.0387 (2.2), 127.0387 (1.6), 111.0436 (0.8), 93.0331 (4.9), 85.0279 (7.2)	5.40	1.096
35.	1-caffeoyl-3-hydroxy-dihydrocaffeoylquinic acid isomer	C_25_H_26_O_13_	533.1301	533.1297 (15.1), 371.0985 (65.9), 353.0875 (4.2), 335.0786 (2.0), 191.0553 (15.4), 179.0341 (9.1), 173.0440 (14.8), 161.0232 (2.4), 135.0439 (100), 111.0437 (1.5), 93.0332 (3.9), 85.0279 (2.7)	5.41	−0.683
36.	4-hydroxy-dihydrocaffeoyl-5-caffeoylquinic acid	C_25_H_26_O_13_	533.1301	533.1248 (25.5), 371.0986 (80.6), 353.0876 (8.1), 191.0551 (10.3), 179.0342 (20.4), 173.0446 (100), 161.0233 (2.0), 135.0439 (68.0), 111.0434 (0.7), 93.0330 (22.3), 85.0282 (1.8)	5.57	−9.874
37.	Dicaffeoylquinic acid-hexoside 1	C_31_H_34_O_17_	677.1723	677.1728 (27.7), 515.1407 (54.6), 353.0870 (5.4), 335.0794 (0.6), 179.0341 (100), 173.0445 (7.2), 161.0235 (3.9), 135.0439 (82.2), 93.0331 (4.3), 85.0277 (0.8)	5.61	0.705
38.	4-caffeoyl-5-hydroxy-dihydrocaffeoylquinic acid	C_25_H_26_O_13_	533.1301	533.1309 (100), 371.0998 (9.5), 353.0882 (19.9), 335.0771 (2.0), 191.0552 (77.2), 179.0341 (48.1), 173.0445 (47.6), 161.0232 (8.5), 135.0438 (48.8), 93.0331 (18.6), 85.0279 (9.7)	5.72	1.606
39.	Dicaffeoylquinic acid-hexoside 2	C_31_H_34_O_17_	677.1723	677.1729 (84.6), 515.1387 (57.7), 353.0877 (23.3), 341.0880 (20.1), 335.0775 (5.0), 323.0770 (15.8), 191.0553 (60.1), 179.0341 (100), 173.0447 (44.5), 161.0233 (21.4), 135.0438 (90.5), 111.0436 (4.5), 93.0329 (16.2), 85.0278 (4.7)	5.99	0.867
40.	Dicaffeoylquinic acid-hexoside 3	C_31_H_34_O_17_	677.1723	677.1726 (100), 515.1225 (18.7), 353.0878 (34.1), 341.0884 (3.8), 335.0791 (2.7), 323.0777 (15.9), 191.0553 (52.8), 179.0341 (65.0), 173.0446 (78.3), 161.0233 (31.0), 135.0439 (67.8), 111.0438 (4.2), 93.0330 (24.0), 85.0278 (6.9)	6.34	0.424
41.	Dicaffeoylquinic acid-hexoside 4	C_31_H_34_O_17_	677.1723	677.1724 (96.0), 515.1360 (52.1), 353.0885 (16.2), 341.0877 (55.3), 323.0768 (3.2), 191.0556 (16.3), 179.0341 (100), 173.0446 (53.8), 161.0236 (7.7), 135.0439 (95.0), 111.0437 (3.0), 93.0330 (23.8), 85.0275 (0.6)	6.46	0.144
42.	3,4-dicaffeoylquinic acid *	C_25_H_24_O_12_	515.1190	515.1197 (100), 353.0877 (15.3), 335.0784 (4.8), 191.0553 (22.9), 179.0341 (47.3), 173.0446 (55.0), 161.0232 (10.5), 135.0438 (27.0), 111.0437 (1.7), 93.0332 (7.4), 85.0277 (2.2)	6.55	0.370
43.	3,5-dicaffeoylquinic acid *	C_25_H_24_O_12_	515.1190	515.1204 (14.8), 353.0880 (94.3), 191.0553 (100), 179.0341 (57.0), 173.0441 (4.0), 161.0237 (4.4), 135.0439 (37.0), 111.0438 (0.7), 93.0331 (3.3), 85.0279 (8.8)	6.80	1.787
44.	4,5-dicaffeoylquinic acid	C_25_H_24_O_12_	515.1190	515.1196 (85.6), 353.0880 (66.0), 335.0795 (0.8), 191.0554 (36.7), 179.0341 (70.8), 173.0446 (100), 135.0439 (68.8), 111.0436 (4.3), 93.0331 (26.5), 85.0280 (5.4)	7.13	0.137
45.	3-*p*-coumaroyl-5-caffeoylquinic acid	C_25_H_24_O_11_	499.1251	499.1242 (19.0), 353.0882 (6.3), 337.0933 (74.6), 191.0553 (17.5), 173.0446 (7.3), 163.0390 (100), 135.0436 (3.9), 119.0488 (34.3), 111.0435 (1.8), 93.0333 (3.3), 85.0276 (1.9)	7.58	−0.691
46.	3-caffeoyl-5-*p*-coumaroylquinic acid	C_25_H_24_O_11_	499.1251	499.1243 (23.2), 353.0883 (58.8), 337.0932 (28.5), 319.0830 (1.2), 191.0554 (100), 179.0342 (37.4), 163.0391 (10.8), 161.0233 (5.7), 135.0439 (31.5), 119.0489 (5.4), 93.0332 (10.8), 85.0280 (5.8)	7.64	−0.510
47.	3-feruloyl-5-caffeoylquinic acid	C_26_H_26_O_12_	529.1356	529.1364 (11.4), 367.1034 (97.6), 335.0771 (1.2), 193.0499 (100), 191.0554 (2.0), 179.0331 (0.8), 173.0445 (9.2), 161.0228 (2.2), 135.0401 (3.0), 134.0360 (67.1), 93.0331 (3.0)	7.84	2.269
48.	1-caffeoyl-5-feruloylquinic acid	C_26_H_26_O_12_	529.1356	529.1355 (31.4), 367.1035 (51.8), 353.0877 (33.7), 335.7119 (0.5), 193.0499 (23.6), 191.0552 (100), 179.0339 (30.9), 173.0445 (13.7), 161.0233 (13.7), 135.0439 (26.0), 134.0360 (22.2), 111.0435 (2.2), 93.0330 (14.0), 85.0280 (5.7)	7.92	0.663
49.	4-*p*-coumaroyl-5-caffeoylquinic acid	C_25_H_24_O_11_	499.1252	499.1248 (27.0), 337.0932 (73.0), 173.0446 (100), 163.0388 (19.1), 135.0436 (4.4), 133.0278 (1.4), 127.0385 (1.4), 121.0279 (45.9), 119.0486 (11.2), 111.0435 (3.3), 93.0329 (24.5), 85.0279 (1.9)	7.93	0.411
50.	4-caffeoyl-5-*p*-coumaroylquinic acid	C_25_H_24_O_11_	499.1252	499.1259 (17.6), 353.0881 (57.4), 337.0937 (10.4), 191.0554 (49.5), 179.0341 (65.9), 173.0445 (100), 135.0439 (55.0), 119.0491 (1.5), 111.0436 (3.5), 93.0331 (27.1), 85.0279 (5.2)	8.01	2.635
51.	4-feruloyl-5-caffeoylquinic acid	C_26_H_26_O_12_	529.1356	529.1354 (19.7), 367.1034 (67.2), 193.0500 (18.6), 173.0446 (100), 161.0234 (1.3), 135.0444 (0.9), 134.0360 (15.7), 111.0439 (4.4), 93.0330 (25.4)	8.11	0.436
52.	4-caffeoyl-5-feruloylquinic acid	C_26_H_26_O_12_	529.1356	529.1353 (90.3), 367.1035 (25.3), 353.0880 (61.2), 335.0771 (1.1), 193.0488 (5.0), 191.0553 (61.2), 173.0446 (100), 161.0233 (11.1), 135.0439 (56.6), 111.0435 (4.6), 93.0330 (31.2), 85.0279 (5.7)	8.18	0.190
53.	1(3)-caffeoyl-4-feruloylquinic acid	C_26_H_26_O_12_	529.1356	529.1351 (100), 367.1034 (37.0), 353.0905 (0.7), 335.0794 (1.1), 193.0503 (6.0), 191.0547 (2.4), 179.0341 (18.6), 173.0445 (46.6), 161.0234 (22.3), 135.0438 (21.8), 134.0361 (12.3), 111.0437 (8.7), 93.0330 (61.3), 85.0279 (0.8)	8.32	−0.037
54.	3,4,5-tricaffeoylquinic acid	C_34_H_30_O_15_	677.1528	677.1517 (100), 515.1191 (41.3), 353.0881 (39.6), 335.0770 (10.2), 191.0554 (41.8), 179.0341 (63.6), 173.0446 (80.8), 161.0233 (25.3), 135.0438 (80.2), 111.0435 (5.0), 93.0330 (22.1), 85.0279 (5.8)	8.80	0.704
Flavonoids						
55.	Naringenin diC-hexoside	C_27_H_32_O_15_	595.1668	595.1672 (100), 475.1219 (3.2), 457.1156 (1.6), 415.1035 (10.7), 385.0933 (30.2), 355.0826 (36.0), 119.0488 (13.7), 151.0027 (1.8), 107.0183 (3.4)	4.29	0.566
56.	Apigenin diC-hexoside	C_27_H_29_O_15_	593.1512	593.1516 (100), 503.1123 (4.2), 473.1091 (14.3), 413.0897 (2.8), 383.0772 (19.2), 353.0668 (34.1), 325.0730 (2.5), 297.0770 (9.6), 283.0616 (0.9), 161.0234 (1.3),	4.80	0.703
57.	Eriodyctiol methyl ether *O*-hexuronide	C_22_H_22_O_13_	493.0988	493.0991 (51.4), 317.0668 (100), 299.0567 (2.2), 289.0721 (6.7), 274.0481 (1.1), 258.0525 (2.1), 175.0240 (10.8), 125.0231 (0.6), 107.0120 (0.8), 151.0026 (5.3)	5.04	0.722
58.	Scutelarein *O*-hexoside	C_21_H_20_O_11_	463.0933	463.0886 (100), 301.0355 (72.5), 300.0276 (26.6), 283.0259 (0.5), 255.0291 (1.6), 243.0294 (1.3), 227.0344 (2.1), 201.0184 (2.2), 139.0023 (1.3), 136.9868 (6.4)	5.69	0.779
59.	Eryodictiol *O*-hexoside	C_21_H_22_O_11_	449.1089	449.1091 (100), 287.0565 (57.2), 175.0238 (13.3), 161.0232 (3.1), 151.0025 (95.2), 135.0439 (64.8), 125.0230 (6.9), 107.0123 (16.4)	6.03	0.435
60.	Rutin	C_27_H_30_O_16_	609.1461	609.1462 (100), 301.0349 (36.8), 300.0276 (67.1), 271.0250 (31.8), 255.0297 (14.1), 243.0296 (5.7), 178.9980 (2.8), 161.0236 (2.6), 151.0027 (7.0), 135.0082 (0.5), 121.0278 (1.1), 107.0123 (2.3)	6.08	0.118
61.	Quercetin *O*-hexuronide	C_21_H_18_O_13_	477.0669	477.0674 (46.9), 373.0573 (0.7), 343.0472 (1.3), 301.0355 (100), 255.0297 (1.3), 227.0343 (1.3), 211.0399 (0.5), 178.9981 (3.1), 163.0026 (0.5), 151.0024 (11.8), 121.0283 (3.3), 107.0124 (3.7)	6.15	−0.050
62.	Luteolin 7-*O*-rutinoside	C_27_H_30_O_15_	593.1507	593.1577 (100), 285.0406 (98.0), 256.0394 (0.6), 241.0500 (0.7), 217.0495 (0.9), 175.0384 (0.9), 151.0020 (3.9), 133.0281 (5.0)	6.16	0.905
63.	Isoquercitrin/hyperoside	C_21_H_20_O_12_	463.0885	463.0887 (100), 301.0348 (44.3), 300.0275 (71.3), 271.0247 (37.4), 255.0296 (16.5), 243.0296 (8.6), 227.0340 (2.8), 178.9973 (2.6), 163.0024 (2.2), 151.0023 (6.0), 121.0275 (1.5), 107.0121 (1.8)	6.20	1.038
64.	Luteolin *O*-hexuronide1	C_21_H_18_O_12_	461.0736	461.0726 (55.3), 357.0613 (0.5), 327.0516 (0.8), 285.0405 (100), 243.0287 (0.6), 175.0382 (2.8), 151.0025 (4.1), 149.0390 (2.2), 133.0025 (7.9), 107.0119 (2.4)	6.33	0.175
65.	Luteolin 7-*O*-glucoside *	C_21_H_20_O_11_	447.0934	447.0934 (100), 327.0517 (1.7), 285.0406 (75.8), 256.0380 (2.3), 227.0342 (1.5), 211.0393 (0.5), 199.0391 (1.1), 175.0394 (0.5), 151.0025 (2.8), 133.0280 (3.0), 107.0121 (1.8)	6.40	0.348
66.	Patuletin *O*-hexoside	C_22_H_22_O_13_	493.0987	493.0977 (100), 331.0461 (78.6), 316.0224 (11.2), 287.0196 (2.5), 271.0254 (1.7), 259.0251 (0.7), 243.0302 (1.1), 227.0370 (0.4), 215.0343 (0.3), 178.9980 (1.3), 165.9898 (9.6), 139.0024 (7.0), 136.9873 (1.0), 109.9995 (4.5)	6.51	−2.239
67.	Nepetin *O*-hexoside	C_22_H_22_O_12_	447.1038	477.1040 (100), 315.0508 (59.9), 314.0433 (25.4), 300.0273 (11.9), 299.1200 (19.0), 271.0247 (18.4), 255.0302 (0.9), 243.0299 (1.8), 199.0396 (5.5), 151.0023 (0.6), 136.9865 (3.4), 133.0278 (0.6)	6.60	0.316
68.	Isoquercitrin/hyperoside	C_21_H_20_O_12_	463.0885	463.0882 (94.5), 301.0358 (100), 271.0256 (2.0), 243.0292 (0.6), 151.0025 (18.1), 107.0124 (9.4)	6.65	0.002
69.	Kaempferol-methyl ether *O*-hexuronide/Nepetin *O*-hexuronide	C_22_H_20_O_13_	491.0832	491.0833 (100), 315.0513 (94.5), 300.0278 (40.1), 271.0249 (47.7), 255.0298 (5.3), 243.029 (1.6), 227.0350 (2.3), 175.024 (2.7), 163.0027 (2.7)	6.68	0.461
70.	Naringenin *O*-hexuronide	C_21_H_20_O_11_	447.0932	447.0934 (69.3), 271.0615 (100), 175.0239 (53.0), 151.0025 (44.6), 119.0489 (35.6), 107.0124 (17.7)	6.98	0.213
71.	Luteolin *O*-acetylhexoside	C_22_H_20_O_12_	489.1038	489.1024 (100), 429.0822 (7.3), 285.0405 (72.9), 267.0288 (0.6), 256.0361 (2.8), 227.0344 (2.7), 211.0397 (2.8), 151.0020 (2.5), 133.0282 (4.8), 107.0121 (0.7)	7.06	−2.983
72.	Luteolin *O*-hexuronide2	C_21_H_18_O_12_	461.0736	461.0726 (25.0), 285.0405 (100), 243.0287 (0.4), 217.0502 (1.1), 199.0393 (2.3), 175.0387 (1.5), 151.0024 (5.6), 133.0281 (13.7), 107.0125 (3.0)	7.08	0.175
73.	Isorhamnetin 3-*O*-glucoside *	C_22_H_22_O_12_	477.1038	477.1040 (100), 357.0620 (0.7), 315.0498 (8.8), 314.0435 (47.7), 299.0199 (4.3), 271.0251 (18.9), 257.0450 (5.3), 243.0296 (20.7), 227.0347 (3.0), 199.0392 (3.3), 178.9973 (0.5), 151.0023 (2.8)	7.08	0.379
74.	Apigenin 7-*O*-glucoside *	C_21_H_20_O_10_	431.0984	431.0983 (100), 311.0562 (0.6), 269.0451 (27.3), 268.0378 (54.7), 211.0399 (2.1), 151.0025 (2.9), 117.0330 (1.8), 107.0121 (1.5)	7.09	−0.162
75.	Apigenin *O*-hexuronide	C_21_H_18_O_11_	445.0787	445.0775 (29.9), 269.0457 (100), 225.0551 (0.7), 175.0239 (17.6), 151.0020 (2.4), 117.0330 (1.9), 107.0122 (3.5)	7.11	0.885
76.	Nepetin *O*-hexuronide	C_22_H_20_O_13_	491.0832	491.0833 52.6), 315.0514 (100), 300.0277 (24.4), 271.0243 (2.7), 255.0303 (1.6), 227.0344 (1.4), 175.0240 (11.3), 151.0025 (11.1), 148.0153 (2.2), 107.0124 (6.6)	7.26	1.516
77.	Hispidulin *O*-hexuronide	C_22_H_20_O_12_	475.0882	475.0881 (84.9), 299.0562 (100), 284.0317 (61.2), 256.0376 (60.7), 227.0326 (1.5), 175.0238 (15.5), 151.0025 (2.8), 107.0122 (1.1)	7.34	−0.209
78.	Isorhamnetin *O*-hexuronide	C_22_H_20_O_13_	491.0832	491.0836 (60.2), 315.0513 (100), 300.0277 (54.9), 271.0261 (2.4), 255.0312 (2.2), 243.0298 (0.7), 227.0349 (2.3), 175.0239 (20.1), 151.0025 (10.9), 107.0124 (6.7)	7.39	0.970
79.	Hesperetin *O*-hexuronide	C_22_H_22_O_12_	477.1038	477.1039 (94.6), 301.0720 (100), 286.0490 (6.2), 175.0238 (32.9), 151.0026 (14.1), 107.0125 (7.7)	7.45	0.106
80.	Chrysoeriol *O*-hexuronide	C_23_H_22_O_12_	475.0882	475.0878 (100), 299.0563 (90.4), 284.0327 (91.1), 256.0372 (6.1), 217.1775 (3.7), 175.0240 (28.2), 151.0024 (3.9), 117.0177 (3.7)	7.55	−0.840
81.	Chrysoeriol *O*-hexoside	C_22_H_22_O_11_	461.1089	461.1119 (16.6), 299.0561 (100), 284.0328 (55.0), 256.0377 (2.4), 227.0339 (0.4), 151.0026 (1.3), 133.0270 (0.9)	7.73	6.518
82.	Jaceosidin/cirsiliol *O*-hexuronide	C_23_H_22_O_13_	505.0988	505.0990 (100), 329.0668 (76.03), 314.0435 (28.4), 299.0199 (53.4), 271.0251 (40.2), 243.0300 (0.79), 227.0341 (2.76), 199.0389 (1.48), 175.0237 (26.8), 113.0230 (48.49), 85.0279 (29.51)	7.89	0.408
83.	Luteolin *O*-caffeoylhexoside	C_30_H_26_O_14_	609.1250	609.1254 (88.3), 447.0908 (2.9), 285.0406 (100), 257.0470 (0.4), 221.0442 (1.6), 199.0388 (2.4), 179.0342 (3.0), 161.0233 (14.0), 151.0024 (4.3), 135.0440 (4.8), 133.0282 (14.6), 121.0274 (0.8)	7.98	0.741
84.	Luteolin *	C_15_H_9_O_7_	285.0406	285.0404 (100), 243.0303 (0.44), 217.0502 (1.29), 199.0390 (2.72), 175.0388 (4.56), 151.0024 (6.36), 133.0282 (35.4), 107.0124 (5.81)	8.84	−0.215
85.	Quercetin *	C_15_H_10_O_7_	301.0354	301.0355 (100), 273.0400 (2.09), 255.0313 (0.45), 229.0500 (1.07), 178.9976 (19.44), 161.0232 (0.95), 151.0875 (37.74), 121.0281 (10.21), 107.0124 (11.2)	8.90	0.279
86.	Nepetin	C_16_H_12_O_7_	315.0510	315.0512 (78.38), 300.0276 (100), 271.0236 (0.69), 243.0297 (1.46), 227.0343 (2.07), 165.9900 (1.37), 136.9867 (10.76), 139.0023 (0.48), 133.0280 (2.28)	9.11	0.553
87.	6-methoxykaempferol	C_16_H_12_O_7_	315.0510	315.0513 (75.4), 300.0276 (100), 271.0249 (37.1), 255.0298 (15.46), 243.0297 (10.4), 227.0346 (1.94), 199.0396 (1.38), 148.0152 (2.46)	9.36	0.775
88.	Axillarin (quercetagetin 3,6-dimethylether)	C_17_H_14_O_8_	345.0616	345.0616 (100), 330.0382 (90.9), 315.0148 (50.7), 287.0197 (8.2), 271.0249 (1.1), 259.0248 (1.0), 241.0155 (0.8), 231.0295 (4.7), 215.0344 (2.7), 165.9898 (2.5), 149.0236 (5.5), 121.0279	9.50	−0.031
89.	Naringenin	C_15_H_12_O_5_	271.0612	271.0614 (100), 227.0707 (0.85), 177.0184 (10.66), 151.0025 (68.65), 145.0283 (1.54), 119.0488 (55.08), 107.0124 (15.02)	9.80	0.639
90.	Apigenin *	C_15_H_10_O_5_	269.0457	269.0457 (100), 225.0553 (1.82), 201.0551 (0.74), 151.0025 (4.63), 117.0331 (13.56), 107.0124 (3.38)	10.15	0.384
91.	Kaempferol *	C_15_H_10_O_6_	285.0405	In (+) ESI-MS/MS:287.0545 (100), 269.0439 (0.5), 258.0513 (1.2), 241.0486 (0.7), 231.0652 (0.3), 213.0542 (1.9), 185.0590 (0.8), 165.0179 (1.8), 153.0181 (7.9), 121.0285 (4.3)	10.29	−1.897
92.	Isorhamnetin *	C_16_H_12_O_7_	315.0510	315.0514 (100), 300.0277 (96.57), 271.0239 (3.65), 255.0295 (2.75), 243.0301 (0.82), 227.0340 (1.34), 199.0397 (0.4), 163.0023 (3.83), 151.0026 (9.54), 107.0124 (9.34)	10.62	1.187
93.	Cirsiliol	C_17_H_14_O_7_	329.0677	329.0668 (93.5), 314.0436 (100), 299.0197 (22.5), 271.0249 (32.1), 255.0285 (0.6), 243.0295 (4.9), 227.0342 (3.0), 215.0342 (2.3), 199.0394 (8.0), 163.0032 (1.1), 136.9857 (10.7), 133.0292 (4.2)	10.72	0.468
94.	Jaceosidin *	C_17_H_14_O_7_	329.0667	329.0668 (100), 314.0435 (46.5), 299.0197 (72.1), 271.0249 (48.4), 255.0296 (0.3), 243.0297 (7.6), 227.0341(4.2), 215.0343 (3.0), 199.0393 (5.4), 175.0387 (1.3), 151.0020 (1.3), 133.0280 (0.6)	11.00	0.377
95.	Quercetagetin-3,6,3′(4′)-trimethyl ether	C_18_H_16_O_8_	359.0772	359.0775 (100), 344.0538 (72.7), 329.0305 (46.1), 314.0063 (2.5), 301.0353 (13.00), 286.0119 (21.8), 258.0171 (14.7), 230.0219 (8.0), 214.0255 (2.1), 202.0265 (9.0), 178.9973 (0.8), 165.9896 (1.8), 163.0387 (0.8)	11.20	0.750
96.	Apigenin dimethyl ether	C_17_H_14_O_6_	313.0718	313.0720 (100), 298.0482 (40.9),283.0249 (15.7), 269.0458 (13.5), 255.0299 (33.9), 225.0553 (0.3), 151.0022 (0.4), 117.0329 (0.5), 107.0123 (0.4)	12.00	0.762
97.	Eupatilin	C_18_H_16_O_7_	343.0812	343.0822 (100), 328.0590 (76.6), 313.0356 (49.9), 298.0122 (19.2), 285.0409 (2.3), 270.0169 (14.1), 242.0214 (4.7), 214.0267 (3.3), 198.0312 (2.5), 163.0027 (5.5), 147.0439 (4.4), 132.0200 (5.5)	12.30	−0.134
98.	Casticin	C_19_H_18_O_8_	373.0929	373.0928 (100), 358.0697 (44.6), 343.0459 (35.1), 328.0218 (4.2), 315.0318 (12.6), 300.0284 (18.9), 285.0041 (24.9), 269.0068 (1.1), 257.0084 (8.2), 241.0143 (1.96), 213.0187 (3.5), 229.0124 (1.56), 201.0177 (1.5)	12.86	−0.163
99.	Galangin methyl ether	C_16_H_12_O_5_	283.0612	283.0612 (100), 268.0378 (67.7), 240.0427 (4.5), 239.0345 (4.5), 171.0440 (1.42), 151.0026 (3.35), 107.0121 (3.08)	13.03	0.188

* Identified by comparison with standard reference.
