# Supplementary material for: Evaluation of the Influence of Tanacetum vulgare Extract on Cognitive Functions and Hippocampal BDNF Expression
Source: Molecules. 2024 Dec 4;29(23):5723. doi: 10.3390/molecules29235723 (PMC11643275; doi:10.3390/molecules29235723)
Supplement: Supplementary file 1 [file molecules-29-05723-s001.zip › molecules-3328635-supplementary.pdf]

Evaluation of the Influence of *Tanacetum vulgare* Extract on Cognitive Functions and Hippocampal BDNF Expression

Borislava Lechkova [1,2], Michaela Shishmanova-Doseva [2,3], Niko Benbassat [1,2], Reneta Gevrenova [4], Pepa Atanassova [5], Nadya Penkova [5], Lyudmil Peychev [3], Petar Hrischev [6], Zhivko Peychev [7] and Stanislava Ivanova [1,2,*]

[1] Department of Pharmacognosy and Pharmaceutical Chemistry, Faculty of Pharmacy, Medical University of Plovdiv, 4002 Plovdiv, Bulgaria; borislava.lechkova@mu-plovdiv.bg (B.L.); niko.benbasat@mu-plovdiv.bg (N.B.)
[2] Research Institute, Medical University of Plovdiv, 4002 Plovdiv, Bulgaria; mihaela.shishmanova@mu-plovdiv.bg
[3] Department of Pharmacology, Toxicology and Pharmacotherapy, Faculty of Pharmacy, Medical University of Plovdiv, 4002 Plovdiv, Bulgaria; lyudmil.peychev@mu-plovdiv.bg
[4] Department of Pharmacognosy, Faculty of Pharmacy, Medical University-Sofia, 2 Dunav Str., 1000 Sofia, Bulgaria; rgevrenova@pharmfac.mu-sofia.bg
[5] Department of Anatomy, Histology and Embryology, Faculty of Medicine, Medical University of Plovdiv, 4002 Plovdiv, Bulgaria; pepa.atanasova@mu-plovdiv.bg (P.A.); nadya.penkova@mu-plovdiv.bg (N.P.)
[6] Department of Physiology, Faculty of Medicine, Medical University of Plovdiv, 4002 Plovdiv, Bulgaria; petar.hrischev@mu-plovdiv.bg
[7] Department of Medical Informatics, Biostatistics and E-Learning, Faculty of Public Health, Medical University of Plovdiv, 4002 Plovdiv, Bulgaria; zhivko.peychev@mu-plovdiv.bg

Supplementary materials

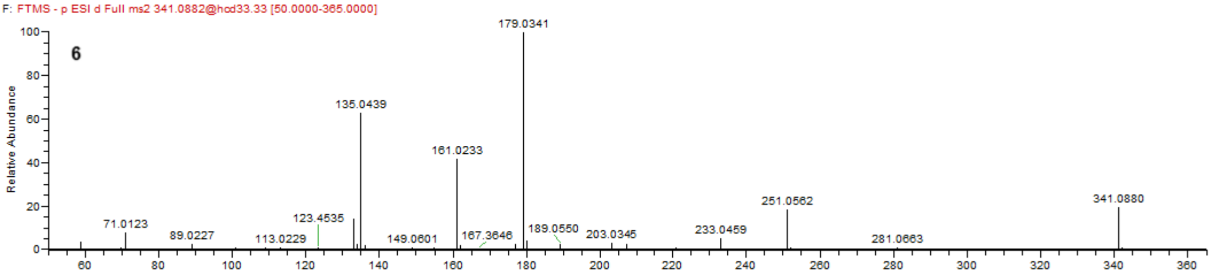

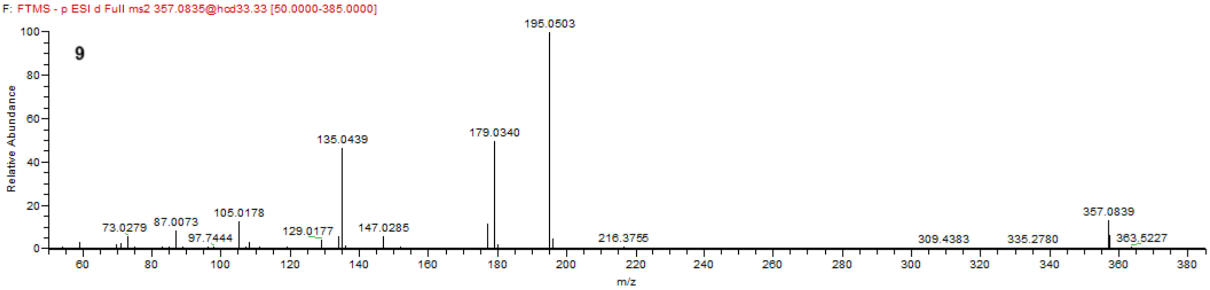

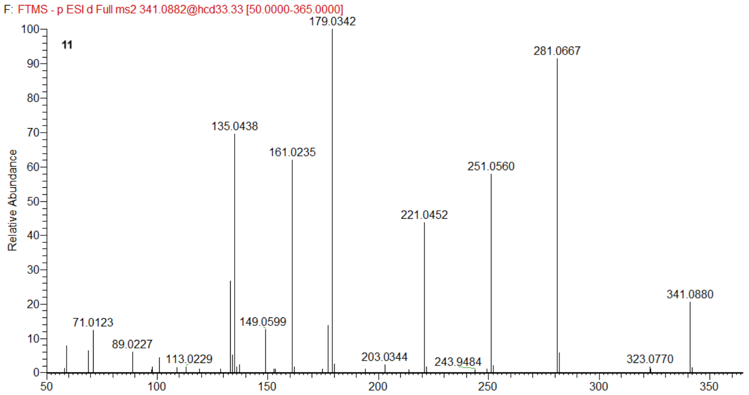

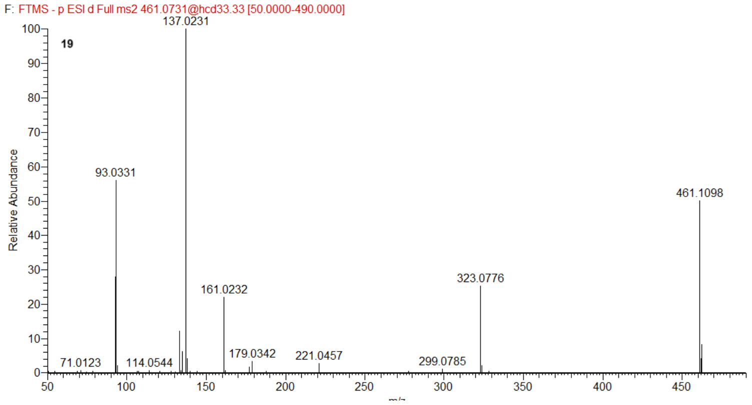

Figure S1. MS/MS spectra of hydroxybenzoic, hydroxycinnamic acids, and their derivatives (for the compound numbers see Table A1).

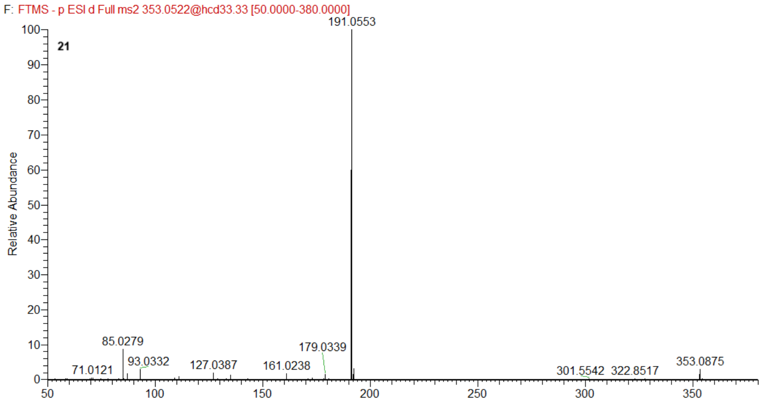

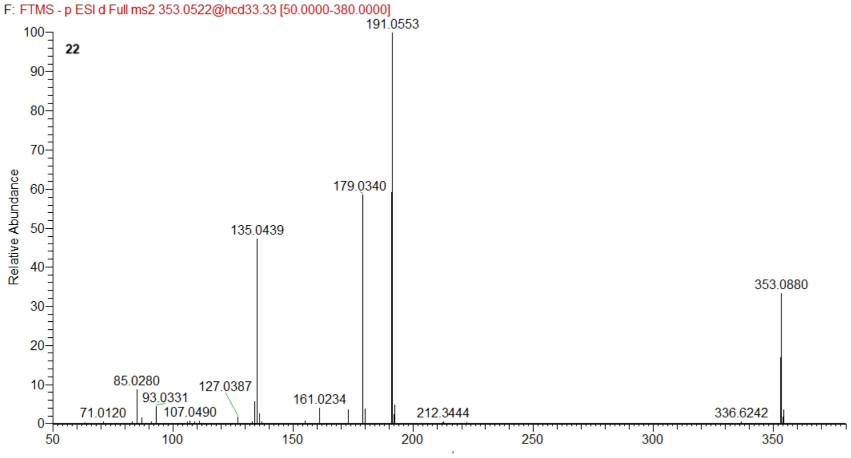

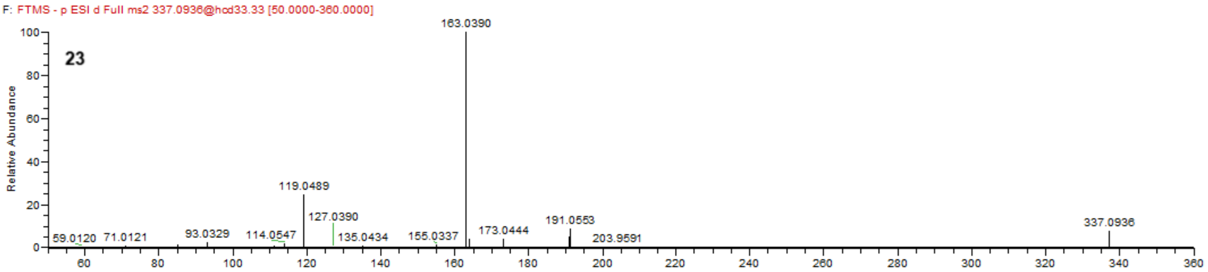
F: FTMS - p ESI d Full ms2 337.0936@hcd33.33 [50.0000-360.0000]
23
Relative Abundance
163.0390
119.0489
127.0390
59.0120
71.0121
93.0329
114.0547
135.0434
155.0337
173.0444
191.0553
203.9591
337.0936

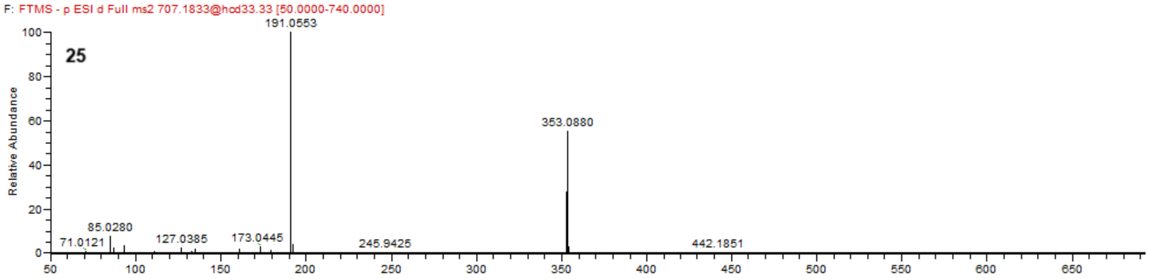
F: FTMS - p ESI d Full ms2 707.1833@hcd33.33 [50.0000-740.0000]
25
Relative Abundance
191.0553
353.0880
85.0280
71.0121
127.0385
173.0445
245.9425
442.1851

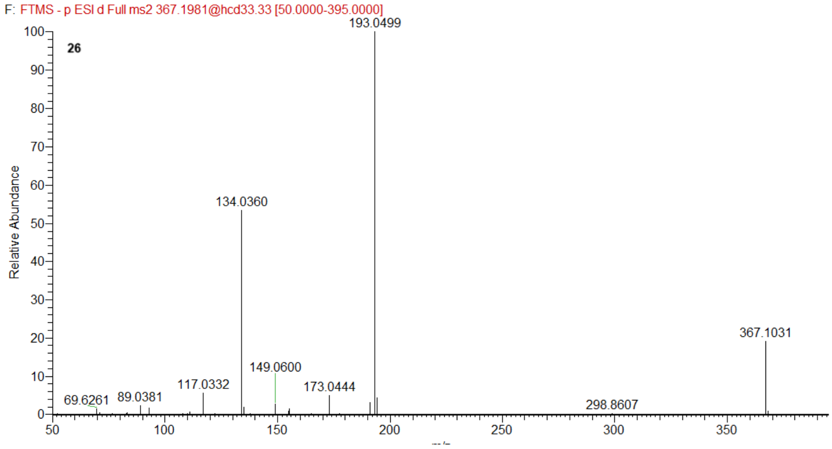
F: FTMS - p ESI d Full ms2 367.1981@hcd33.33 [50.0000-395.0000]
26
Relative Abundance
193.0499
134.0360
367.1031
149.0600
117.0332
173.0444
69.6261
89.0381
298.8607

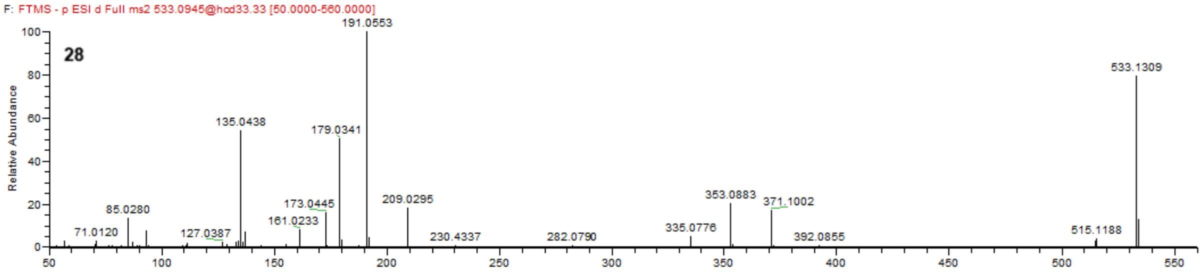
F: FTMS - p ESI d Full ms2 533.0945@hcd33.33 [50.0000-560.0000]
28
Relative Abundance
191.0553
533.1309
135.0438
179.0341
85.0280
173.0445
161.0233
209.0295
353.0883
371.1002
71.0120
127.0387
230.4337
282.0790
335.0776
392.0855
515.1188

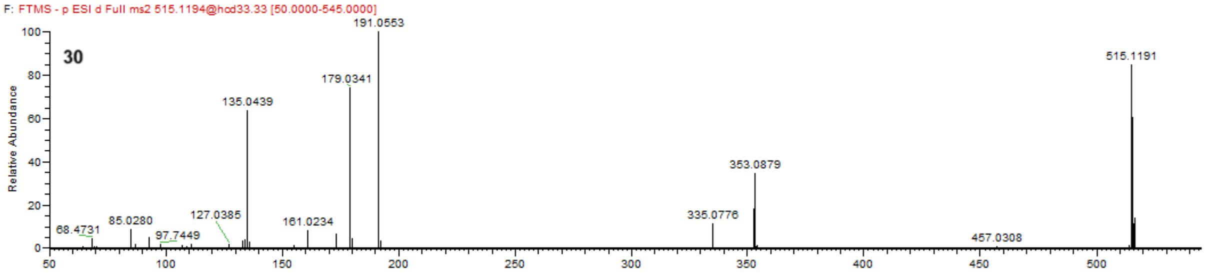
F: FTMS - p ESI d Full ms2 515.1194@hcd33.33 [50.0000-545.0000]
30
Relative Abundance
191.0553
515.1191
179.0341
135.0439
353.0879
335.0776
85.0280
127.0385
161.0234
68.4731
97.7449
457.0308

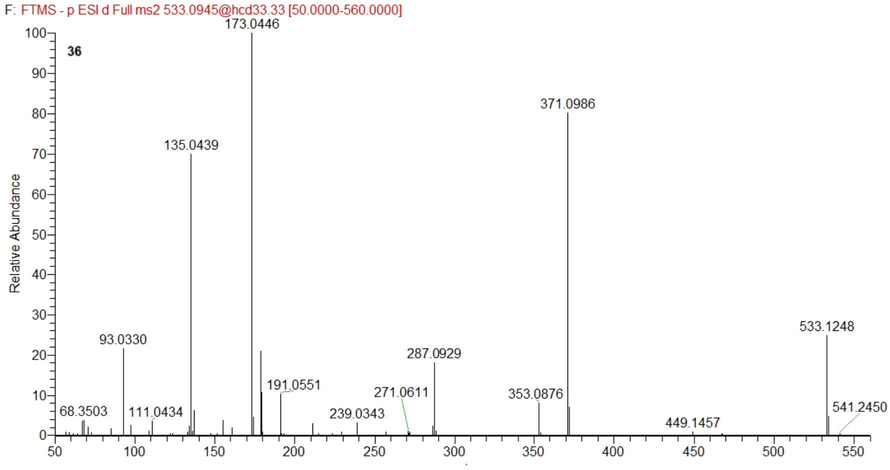
F: FTMS - p ESI d Full ms2 533.0945@hcd33.33 [50.0000-560.0000]
36
Relative Abundance
173.0446
371.0986
135.0439
533.1248
93.0330
287.0929
191.0551
271.0611
353.0876
68.3503
111.0434
239.0343
541.2450
449.1457

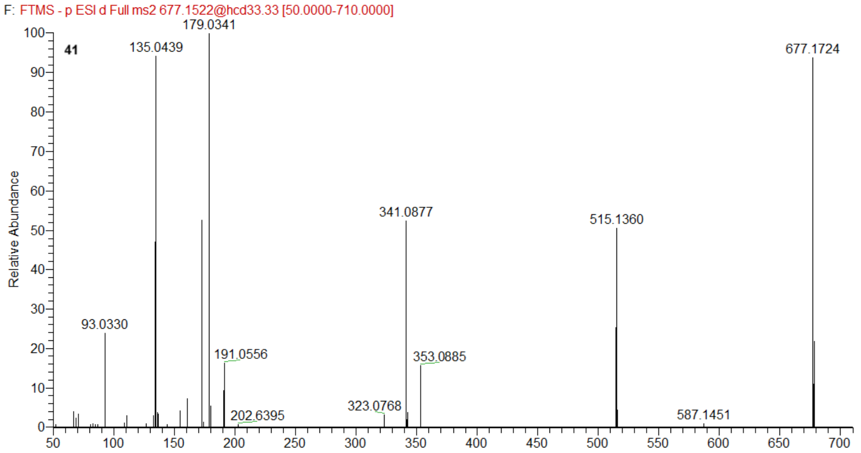
F: FTMS - p ESI d Full ms2 677.1522@hcd33.33 [50.0000-710.0000]
41
Relative Abundance
179.0341
135.0439
677.1724
341.0877
515.1360
93.0330
191.0556
353.0885
323.0768
202.6395
587.1451

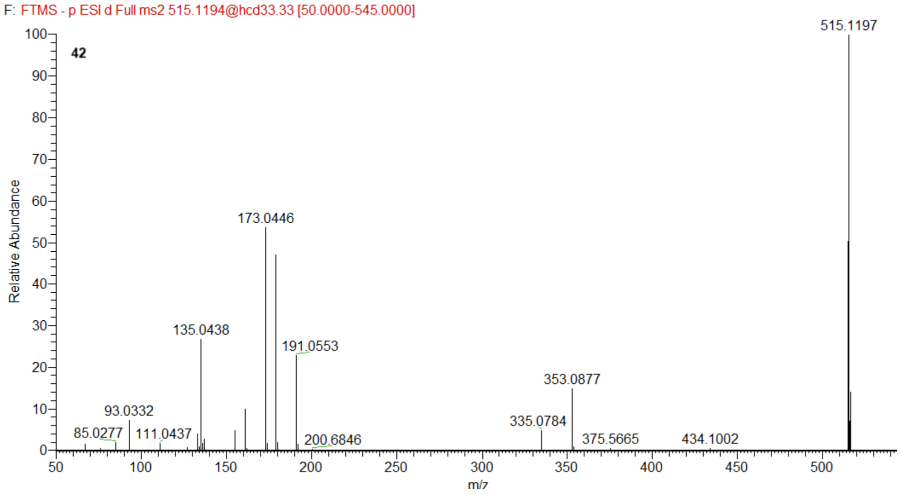
F: FTMS - p ESI d Full ms2 515.1194@hcd33.33 [50.0000-545.0000]
42
Relative Abundance
515.1197
173.0446
135.0438
191.0553
353.0877
93.0332
335.0784
85.0277
111.0437
200.6846
375.5665
434.1002
m/z

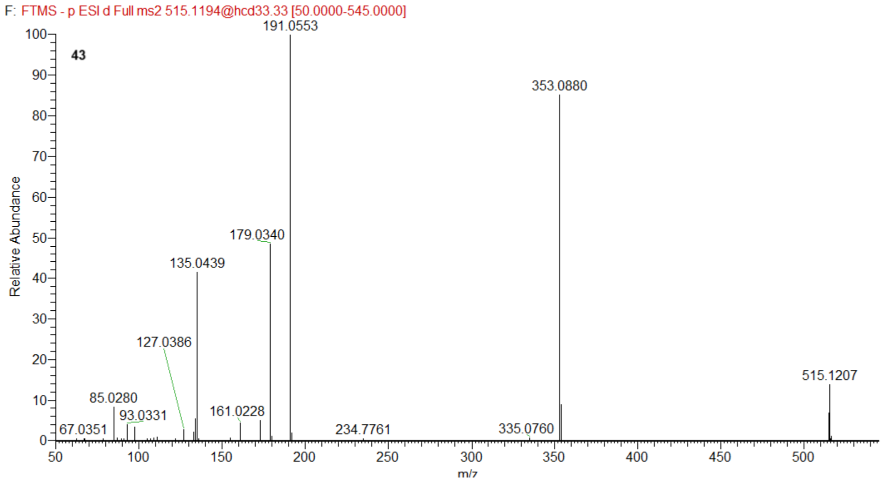
F: FTMS - p ESI d Full ms2 515.1194@hcd33.33 [50.0000-545.0000]
43
Relative Abundance
191.0553
353.0880
179.0340
135.0439
127.0386
85.0280
93.0331
161.0228
67.0351
234.7761
335.0760
515.1207
m/z

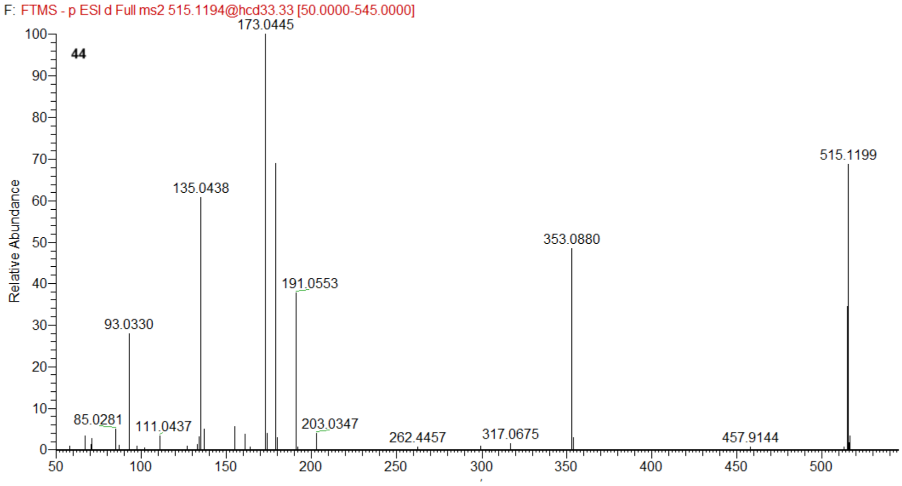
F: FTMS - p ESI d Full ms2 515.1194@hcd33.33 [50.0000-545.0000]
44
Relative Abundance
173.0445
515.1199
135.0438
353.0880
191.0553
93.0330
85.0281
111.0437
203.0347
262.4457
317.0675
457.9144

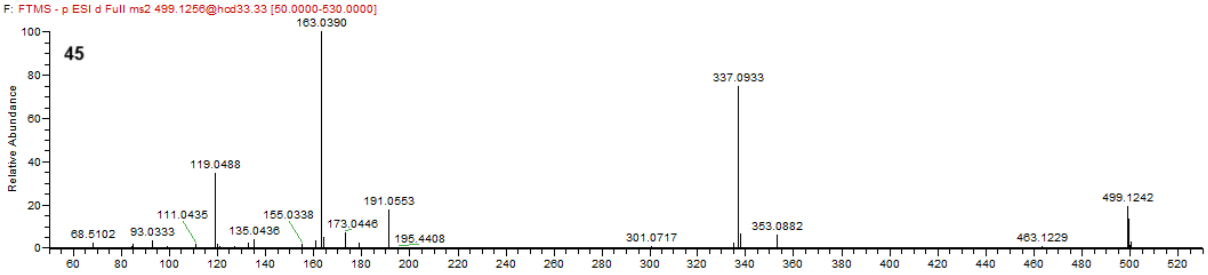
F: FTMS - p ESI d Full ms2 499.1256@hcd33.33 [50.0000-530.0000]
45
Relative Abundance
163.0390
337.0933
119.0488
191.0553
499.1242
111.0435
155.0338
173.0446
68.5102
93.0333
135.0436
195.4408
301.0717
353.0882
463.1229

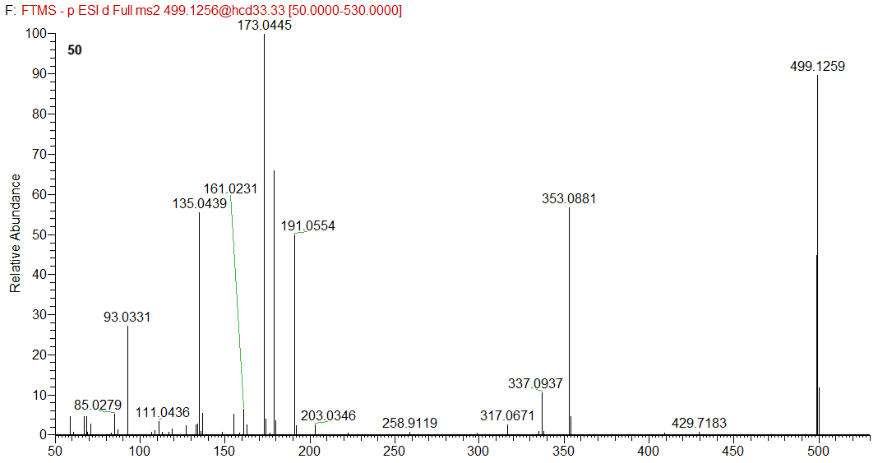
F: FTMS - p ESI d Full ms2 499.1256@hcd33.33 [50.0000-530.0000]
50
Relative Abundance
173.0445
499.1259
161.0231
135.0439
353.0881
191.0554
93.0331
337.0937
85.0279
111.0436
203.0346
258.9119
317.0671
429.7183

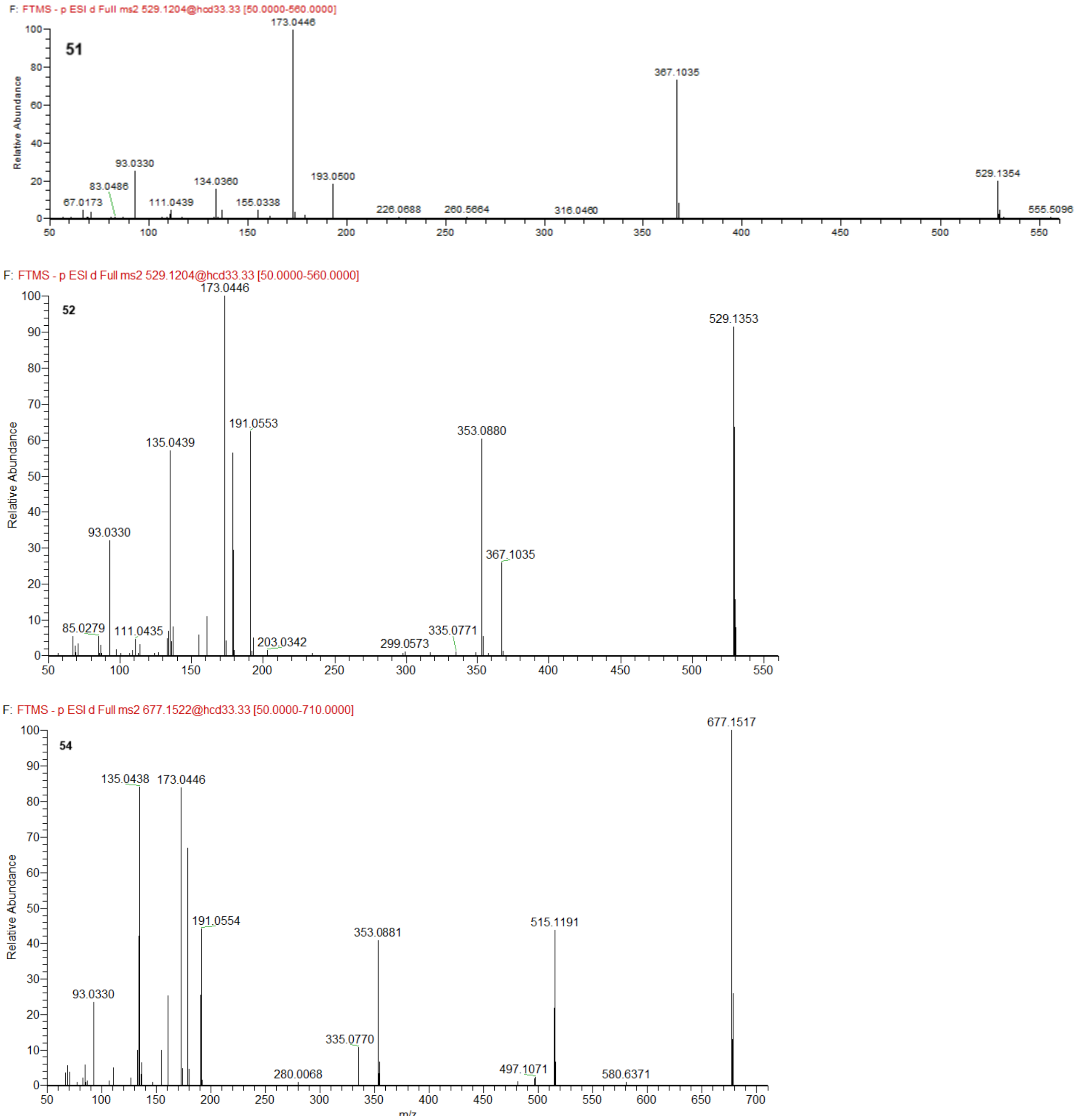

Figure S2. MS/MS spectra of acylquinic acids (for the compound numbers see Table A1).

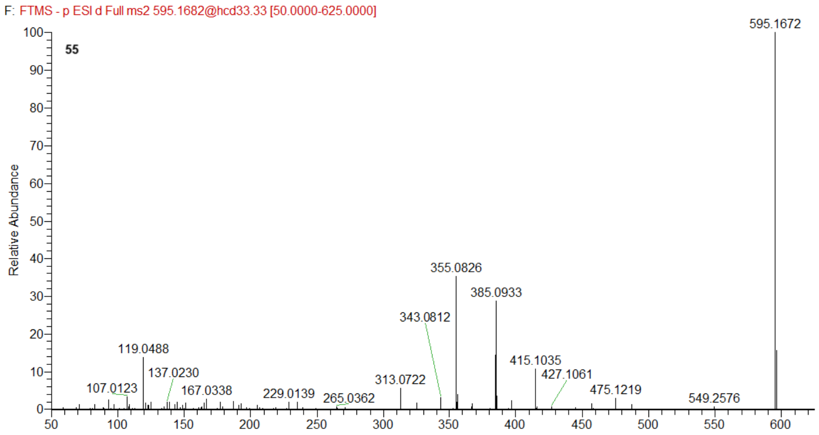
F: FTMS - p ESI d Full ms2 595.1682@hcd33.33 [50.0000-625.0000]
55
Relative Abundance
595.1672
355.0826
385.0933
343.0812
119.0488
415.1035
137.0230
427.1061
313.0722
107.0123
167.0338
229.0139
475.1219
265.0362
549.2576

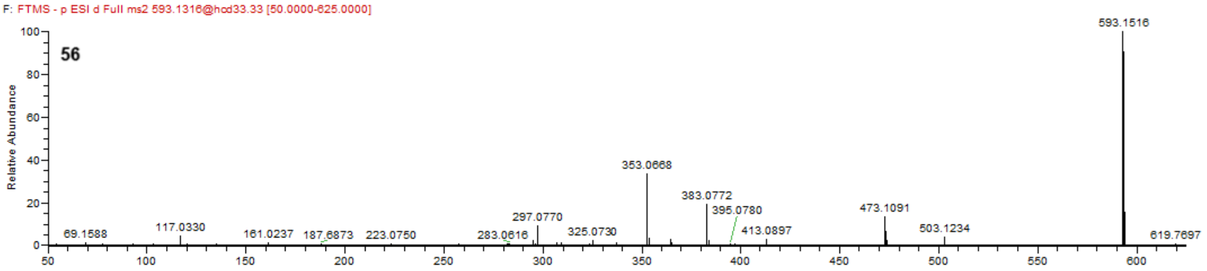
F: FTMS - p ESI d Full ms2 593.1316@hcd33.33 [50.0000-625.0000]
56
Relative Abundance
593.1516
353.0668
383.0772
395.0780
297.0770
473.1091
69.1588
117.0330
161.0237
187.6873
223.0750
283.0616
325.0730
413.0897
503.1234
619.7697

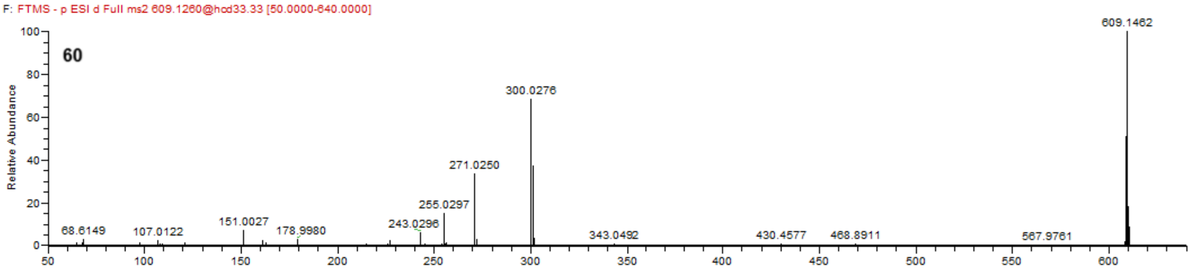
F: FTMS - p ESI d Full ms2 609.1260@hcd33.33 [50.0000-640.0000]
60
Relative Abundance
609.1462
300.0276
271.0250
255.0297
243.0296
151.0027
68.6149
107.0122
178.9980
343.0492
430.4577
468.8911
567.9761

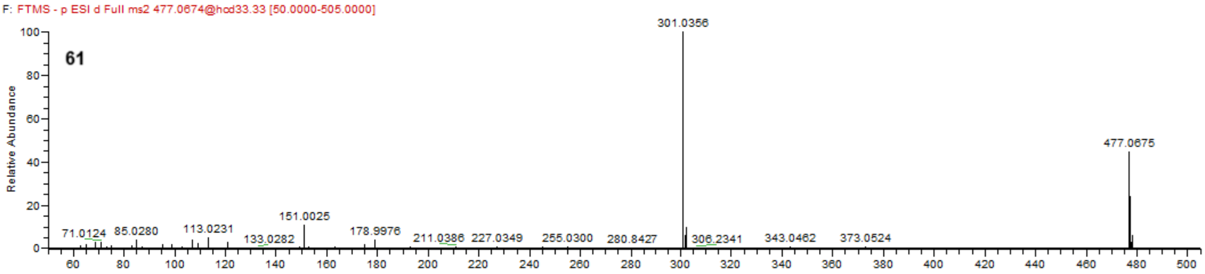
F: FTMS - p ESI d Full ms2 477.0674@hcd33.33 [50.0000-505.0000]
61
Relative Abundance
301.0356
477.0675
151.0025
71.0124
85.0280
113.0231
133.0282
178.9976
211.0386
227.0349
255.0300
280.8427
306.2341
343.0462
373.0524

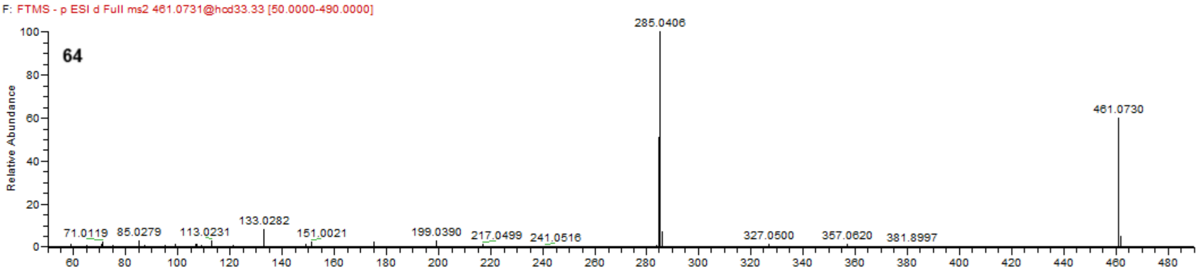
F: FTMS - p ESI d Full ms2 461.0731@hcd33.33 [50.0000-490.0000]
64
Relative Abundance
285.0406
461.0730
133.0282
71.0119
85.0279
113.0231
151.0021
199.0390
217.0499
241.0516
327.0500
357.0620
381.8997

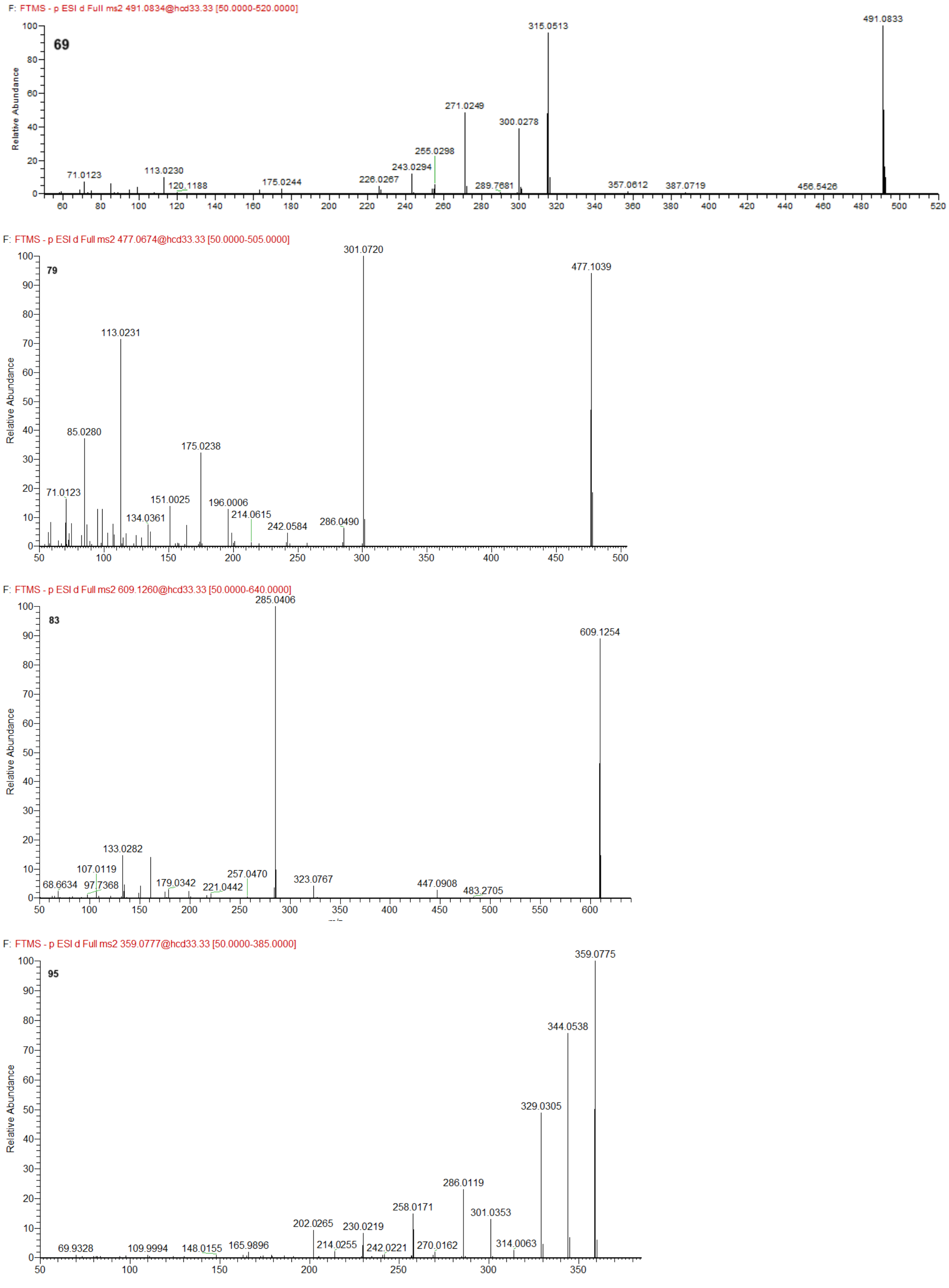

Figure S3. MS/MS spectra of flavonoids (for the compound numbers see Table A1).
